# Profiling Systemic IgG-Targeted Gut Microbiota Reveals Disease Activity-Associated Bacterial Signatures in Ulcerative Colitis

**DOI:** 10.3390/ijms27209081

**Published:** 2026-10-13

**Authors:** Koichi Takahashi, Haruyuki Nakayama-Imaohji, Ayano Tada, Munyeshyaka Emmanuel, Yasuaki Mino, Hideki Ishikawa, Tomomi Kuwahara

**Affiliations:** 1 Department of Microbiology, Faculty of Medicine, Kagawa University, 1750-1 Miki-cho, Kita-gun, Kagawa 761-0793, Japan; takahashi.koichi@kagawa-u.ac.jp (K.T.); imaoji.haruyuki@kagawa-u.ac.jp (H.N.-I.); tada.ayano@kagawa-u.ac.jp (A.T.); emmyshya@gmail.com (M.E.); 2 Department of Pharmacy, Kagawa University Hospital, 1750-1 Miki, Kagawa 761-0793, Japan; 3 Department of Molecular-Targeting Prevention, Graduate School of Medical Science, Kyoto Prefectural University of Medicine, 3-2-17-2F Imabashi, Chuo-ku, Osaka 541-0042, Japan

**Keywords:** IgG+ probability score, ulcerative colitis, gut microbiota, 16S rRNA gene analysis

## Abstract

Aberrant systemic IgG responses to the gut microbiota may be implicated in the pathogenesis of ulcerative colitis (UC). In this study, we investigated IgG-targeted bacteria and their predicted functional characteristics in UC. Using fecal samples and autologous sera from 37 patients with UC and 4 healthy controls, we profiled IgG-targeted bacteria under in vitro conditions using 16S rRNA gene sequencing. To correct for background bacterial abundance, we calculated the IgG+ Probability Score (IPS), which integrates the sample-specific IgG-binding rate to isolate candidate IgG-targeted taxa. Systemic IgG responses were selectively targeted at specific bacterial taxa rather than uniformly directed toward the entire gut microbiota. Active UC and elevated systemic inflammatory markers were associated with increased IPS in oral-associated commensals, including *Granulicatella*, as well as mucin-degrading taxa. Predictive functional profiling indicated the predicted enrichment of pathways hypothesized to be related to host-derived mucin degradation, capsular polysaccharide biosynthesis, and amino acid biosynthesis. Furthermore, exploratory Random Forest models using IPS features highlighted candidate taxa that differed among clinical parameters (UC exacerbation, Clinical Activity Index, and C-reactive protein), although their classification performance was not independently validated. In conclusion, our in vitro profiling indicates that systemic IgG in UC has the capacity to selectively target specific bacterial taxa associated with disease activity and systemic inflammation. Analyzing the IgG-targeted microbiota via IPS profiling deepens our understanding of host–microbiota interactions in UC and provides a valuable framework for evaluating candidate immune-targeted bacteria associated with disease activity.

## 1. Introduction

Inflammatory bowel disease (IBD) is a refractory condition characterized by chronic inflammation of the intestinal mucosa, with alternating periods of relapse and remission. Its pathogenesis is considered to be multifactorial, involving aberrant host immune responses to changes in the gut microbiota (dysbiosis), driven by genetic predisposition and environmental factors such as diet, smoking, and stress [1]. The prevalence of ulcerative colitis (UC), a major phenotype of IBD, continues to increase globally [2,3,4]. In Japan, over 310,000 patients were reported as of 2023, representing a critical public health issue [5]. Current therapeutic strategies primarily focus on inducing and maintaining remission using 5-aminosalicylic acid, corticosteroids, immunosuppressants, and biologics; however, a curative treatment has yet to be established [6,7].

The disruption of the intestinal mucosal barrier is deeply implicated in the pathophysiology of UC [8,9]. Compromised barrier function facilitates the approximation and translocation of luminal bacteria into the mucosa, thereby inducing excessive inflammatory responses. Impaired mucosal barrier function is associated with alterations in gut microbial composition and function. More recently, the relationship between the oral and gut microbiota, known as the oral–gut axis, has attracted considerable attention. Hajishengallis et al. reported that oral-derived bacteria can migrate to the gut via saliva and establish ectopic colonization, potentially contributing to intestinal inflammation and endotoxemia [10,11]. Moreover, periodontitis and IBD are considered to share a common pathophysiological foundation as chronic inflammatory diseases [12,13], and a growing body of evidence supports their epidemiological and immunological associations [14,15,16,17,18,19,20,21,22].

The breakdown of intestinal mucosal homeostasis is accompanied by dramatic alterations in humoral immune responses against the gut microbiota. In the healthy gut, secretory IgA plays a pivotal role in the compartmentalization of luminal bacteria and the maintenance of mucosal homeostasis [23,24,25,26]. Conversely, in UC, the collapse of this immune tolerance leads to aberrant IgA and IgG responses targeting the commensal microbiota. It is well documented that the proportion of IgA- and IgG-coated bacteria in the feces of IBD patients significantly increases during the active phase compared to remission [27]. Furthermore, flow cytometric analyses of fecal samples from IBD patients have demonstrated that the expansion of these IgA- and IgG-coated bacterial populations dynamically correlates with disease severity and clinical course [28]. In particular, the potential involvement of bacteria targeted by systemic IgG responses in IBD pathogenesis has become a major focus of interest. In UC patients, specific IgG responses to commensal bacteria are markedly enhanced, and the resulting IgG-bacteria immune complexes drive pro-inflammatory cytokine production and Th17 responses via macrophage Fcγ receptors [29]. Additionally, IgG-coated bacteria are characteristically observed in IBD patients, and the proportion of bacteria coated by specific IgG subclasses positively correlates with disease activity [30]. These findings suggest that IgG-coated bacteria are not merely a byproduct of barrier dysfunction but actively drive the inflammatory process itself.

Recent advances in IgG-seq technology have enabled the comprehensive profiling of antibody-targeted bacterial populations. Studies utilizing this approach have revealed that IBD patients exhibit pronounced serum IgG responses against specific gut bacterial genera [31]. Moreover, profiling the systemic antimicrobial IgG repertoire has successfully identified specific bacterial taxa capable of breaching the intestinal barrier and translocating into tissues (translocators) [32]. These reports underscore that analyzing bacterial targets of systemic IgG provides crucial insights into host–microbe interactions in IBD.

However, comprehensive analyses of antibody-coated bacteria, including conventional IgG-seq, remain susceptible to confounding by bacterial abundance. When evaluating only the relative abundance within the IgG-bound fraction, highly prevalent taxa that simply increase during inflammation may be overestimated, regardless of their true affinity for IgG [33]. Consequently, the importance of normalization using unfractionated (input) samples to correct for this abundance bias has been advocated [34]. Recently, Jackson et al. and Olm et al. have also highlighted the necessity of incorporating pre-sorted input fractions into downstream analyses [33,35]. These approaches serve as highly valuable frameworks for assessing the relative enrichment of taxa within the IgG-bound fraction while mitigating the influence of dominant commensals in the gut environment [36].

In the present study, we profiled bacterial populations targeted by systemic IgG utilizing an in vitro assay with feces and autologous sera derived from UC patients and healthy controls. To correct for the confounding influence of baseline bacterial abundance, we introduced the IgG+ Probability Score (IPS), a metric that mathematically integrates the sample-specific IgG binding rate to isolate taxa targeted by systemic IgG. Using this framework, we aimed to identify candidate IgG-targeted taxa and evaluate their associations with disease activity and systemic inflammation in UC.

## 2. Results

### 2.1. Clinical Characteristics of the UC Cohort and Evaluation of Autologous Serum IgG Binding Rates

To elucidate the relationship between host antimicrobial antibody responses directed against the gut microbiota and clinical characteristics in patients with UC, we utilized fecal samples and autologous sera collected from 37 patients with UC (24 in the active phase and 13 in remission) and 4 healthy participants (HPs) (Table 1 and Table S1). To determine the reactivity of autologous serum IgG with the gut microbiota (IgG binding rate), 4% paraformaldehyde-fixed fecal samples were incubated with autologous patient sera in vitro and subsequently stained with an APC-conjugated anti-human IgG Fc antibody. The proportion of IgG-bound gut bacteria was then quantified via flow cytometry (Figure S1). The results revealed considerable inter-individual variability in overall IgG binding rates.

Group-level comparisons did not demonstrate statistically significant differences among patients with active UC, patients with remission UC, and HPs (Figure S2A–C). These data indicate that total IgG reactivity to the gut microbiota is highly heterogeneous among individuals.

**Figure 1:**
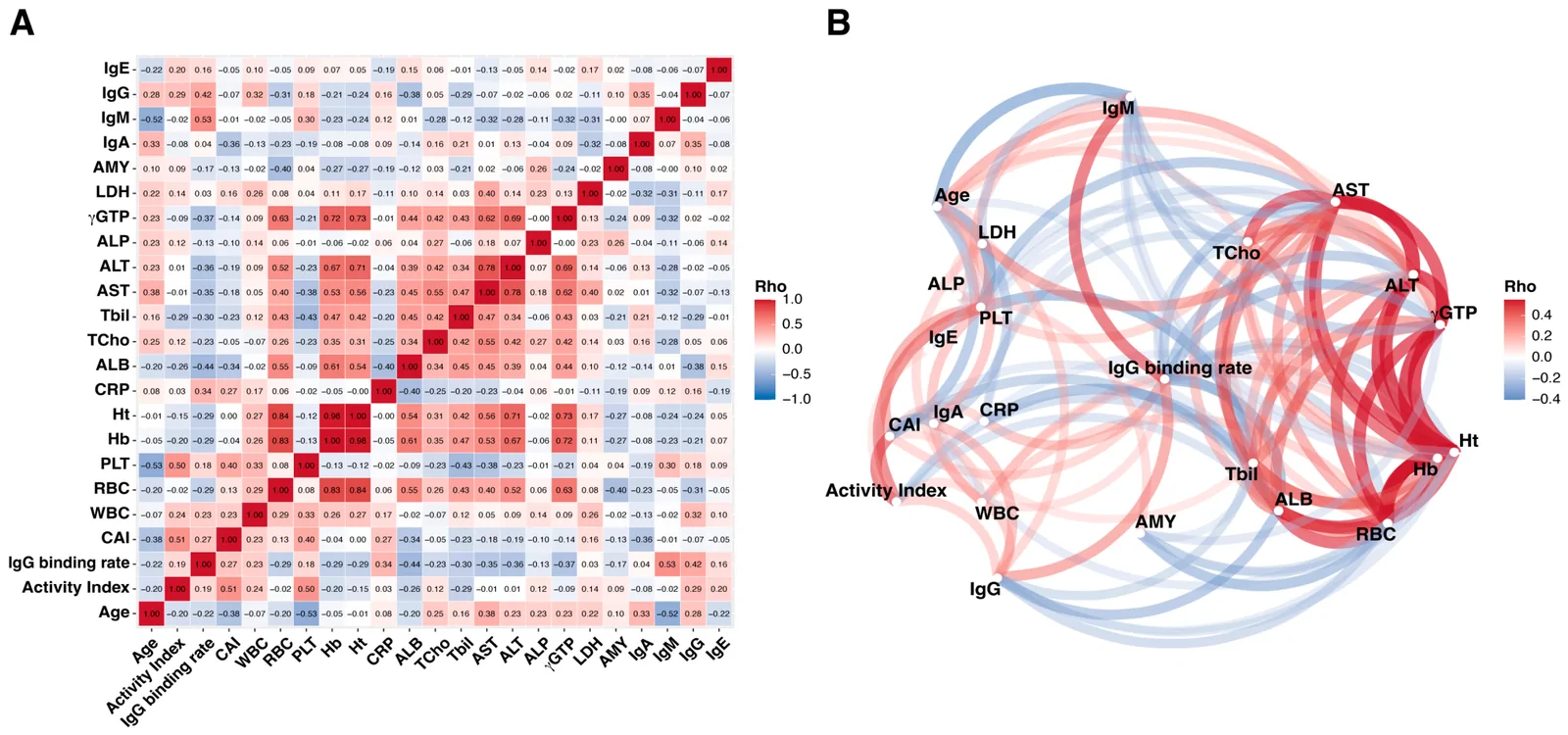
Correlation analysis between clinical parameters and autologous serum IgG binding rates against the gut microbiota. (**A**) Heatmap displaying Spearman’s rank correlation coefficients (Rho) between various clinical parameters and the autologous serum IgG binding rate across the study cohort (*n* = 41). Values within the cells represent the corresponding correlation coefficients. The evaluated parameters include an Activity Index stratifying disease severity (Healthy = 1, Remission = 2, Active = 3). Cell colors indicate the direction and magnitude of the correlations: red represents positive correlations and blue represents negative correlations, with color intensity reflecting the strength of the association. (**B**) Correlation network plot of the clinical parameters. Nodes represent individual clinical parameters, and edges indicate pairwise correlations between variables (displayed only for |Rho| > 0.2). Edge colors (red lines for positive; blue lines for negative) and line thickness correspond to the magnitude of the correlation coefficients. The network demonstrates that disease activity-associated parameters, including the IgG binding rate, form a highly interconnected cluster centered on markers of clinical activity and systemic inflammation, such as CAI and CRP. Abbreviations: CAI, Clinical Activity Index; CRP, C-reactive protein; WBC, White blood cell; RBC, Red blood cell; PLT, Platelets; Hb, Hemoglobin; Ht, Hematocrit; ALB, Albumin; Tcho, Total cholesterol; Tbil, Total bilirubin; AST, Aspartate aminotransferase; ALT, Alanine aminotransferase; ALP, Alkaline phosphatase; γGTP, γ-glutamyl transpeptidase; LDH, Lactate dehydrogenase; AMY, Amylase.

To further investigate the relationship between clinical characteristics and the IgG binding rate, we performed a correlation network analysis incorporating the expanded panels of clinical parameters. The analysis demonstrated that systemic inflammatory markers, including the Clinical Activity Index (CAI), C-reactive protein (CRP), white blood cell (WBC) count, and platelet (PLT) count, exhibited positive correlations with one another, with the IgG binding rate parallel to these indices of disease activity (Figure 1A,B).

Conversely, these inflammatory and disease activity indices showed inverse correlations with hemoglobin (Hb), hematocrit (Ht), red blood cell (RBC) count, and albumin (ALB) levels. Robust positive correlation clusters were also observed among liver-associated enzymes, specifically aspartate aminotransferase (AST), alanine aminotransferase (ALT), γ-glutamyl transpeptidase (γGTP), and alkaline phosphatase (ALP). Additionally, the IgG binding rate on gut microbiota displayed positive correlations with serum immunoglobulin levels, including IgG, IgA, and IgM. These findings indicate that the host humoral immune response directed against the gut microbiota is integrated into a broader network of systemic inflammation, anemia, and altered protein metabolism characteristic of UC pathogenesis. Because the IgG binding rate was positively associated with continuous markers of clinical disease activity (assessed by the CAI) and systemic inflammation (assessed by CRP), it suggests that while overall binding quantities vary, taxon-specific IgG recognition patterns may differ between disease phases, prompting our subsequent taxon-level profiling.

### 2.2. Microbiome Diversity and Structure in Feces and IgG-Bound Fractions

**Figure 2:**
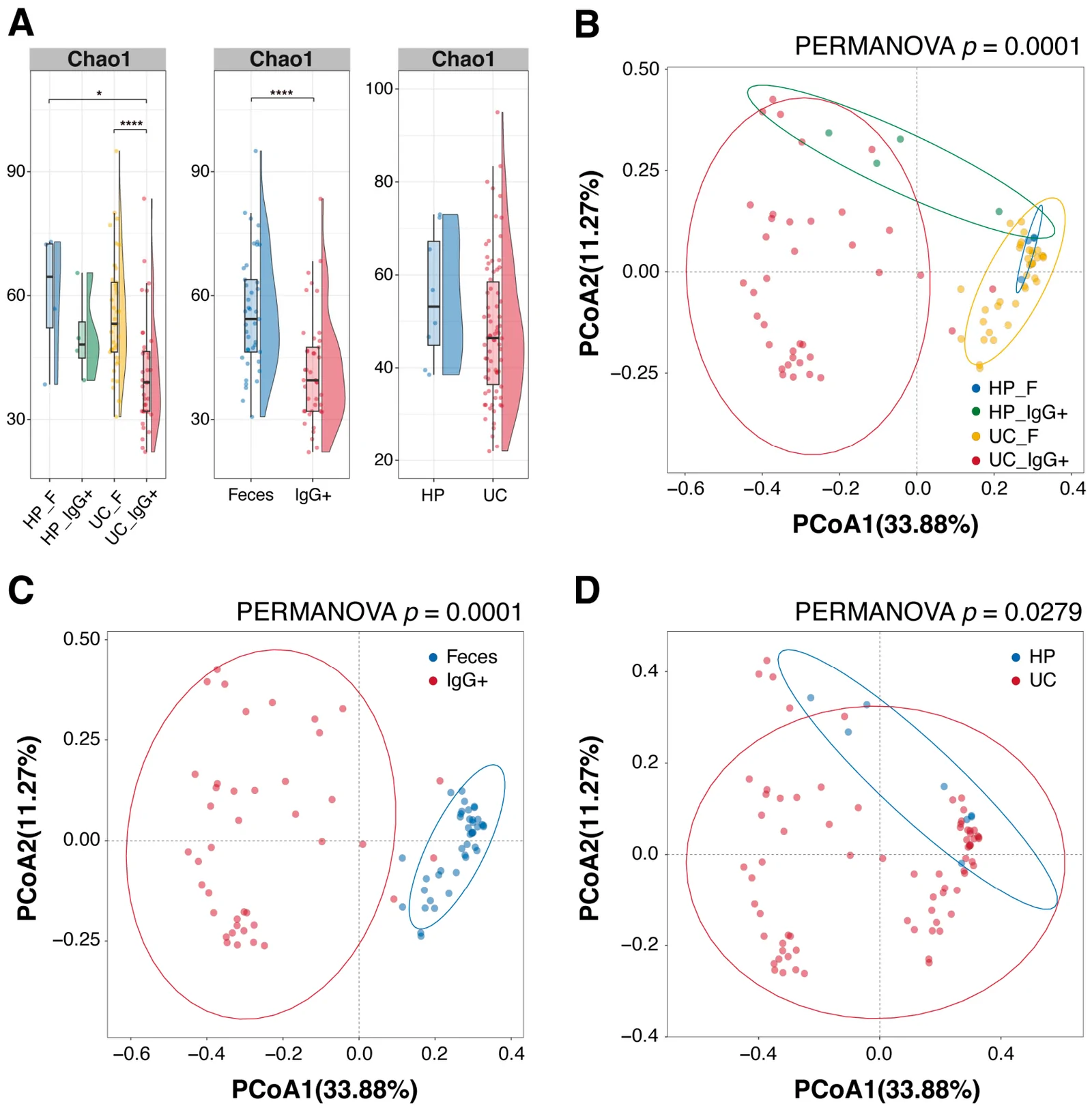
Comparative analysis of gut microbiota diversity and community structure between the feces and autologous serum IgG-bound fractions. (**A**) Comparison of α-diversity, assessed via the Chao1 index, among healthy participants (HP_F: healthy feces fraction, *n* = 4; HP_IgG+: healthy IgG-bound fraction, *n* = 4) and patients with ulcerative colitis (UC_F: UC feces fraction, *n* = 37; UC_IgG+: UC IgG-bound fraction, *n* = 37). Data are presented as violin plots and box plots (indicating the median and interquartile range), overlaid with dots representing individual sample measurements. Statistical significance between groups was evaluated using the Wilcoxon test (Wilcoxon signed-rank test for paired comparisons between fractions, and Wilcoxon rank-sum test for independent group comparisons). Asterisks denote statistical significance (* *p* < 0.05, **** *p* < 0.0001). (**B**–**D**) Principal coordinate analysis (PCoA) plots illustrating the β-diversity of the gut microbiota. The *p*-values within each plot indicate the results of statistical testing for differences in community structure using permutational multivariate analysis of variance (PERMANOVA). The circles indicate the 95% confidence ellipse for each group, respectively. (**B**) Comparison of community structure between the fractions (Feces vs. IgG+) across all samples (total *n* = 82) from healthy participants (HP_F: *n* = 4, HP_IgG+: *n* = 4) and patients with UC (UC_F: *n* = 37, UC_IgG+: *n* = 37). Each dot is color-coded according to the disease group (HP or UC) and fraction type (Feces or IgG+). (**C**) Comparison of community structure between the feces and IgG-bound fractions (Feces vs. IgG+). Each dot is color-coded according to the fraction type (Feces or IgG+). (**D**) Comparison of community structure based on disease status (HP vs. UC). Each dot is color-coded according to the disease group (HP or UC).

We performed α- and β-diversity analyses to compare the ecological characteristics of the microbiota in the feces and IgG-bound (IgG+) fractions. First, to evaluate intra-community species diversity, α diversity was analyzed using the Chao1 index (Figure 2A). In the overall analysis, the α diversity of the IgG+ fraction was significantly lower than that of the feces fraction (*p* < 0.0001). Similar trends were observed within both the HP and UC groups, with a significant decrease in diversity confirmed in the IgG+ fraction (HP group: *p* < 0.05; UC group: *p* < 0.0001). These results indicate that the entire fecal microbiota is not uniformly recognized by IgG; rather, a relatively restricted subset of bacterial taxa is selectively enriched in the IgG-bound fraction. Furthermore, intergroup comparisons revealed a trend toward decreased diversity in the UC group compared to the HP group.

We next analyzed β-diversity using principal coordinate analysis (PCoA) and PERMANOVA to assess differences in overall community structure (Figure 2B–D). The results demonstrated that the feces and IgG+ fractions formed clearly distinct clusters, indicating a significant difference in community structure between the two fractions (*p* = 0.0001, Figure 2B,C). Additionally, an exploratory comparison based on disease status indicated differences in the microbiota structure between the small HP cohort and the UC group (*p* = 0.0279, Figure 2D). Taken together, these findings suggest that the IgG-bound fraction exhibits a microbiota structure distinct from that of the whole feces, reflecting the characteristics of the specific bacterial populations selectively targeted by host IgG.

### 2.3. Profiling of Clinically Associated Bacteria in Fecal and IgG-Bound Fractions

**Figure 3:**
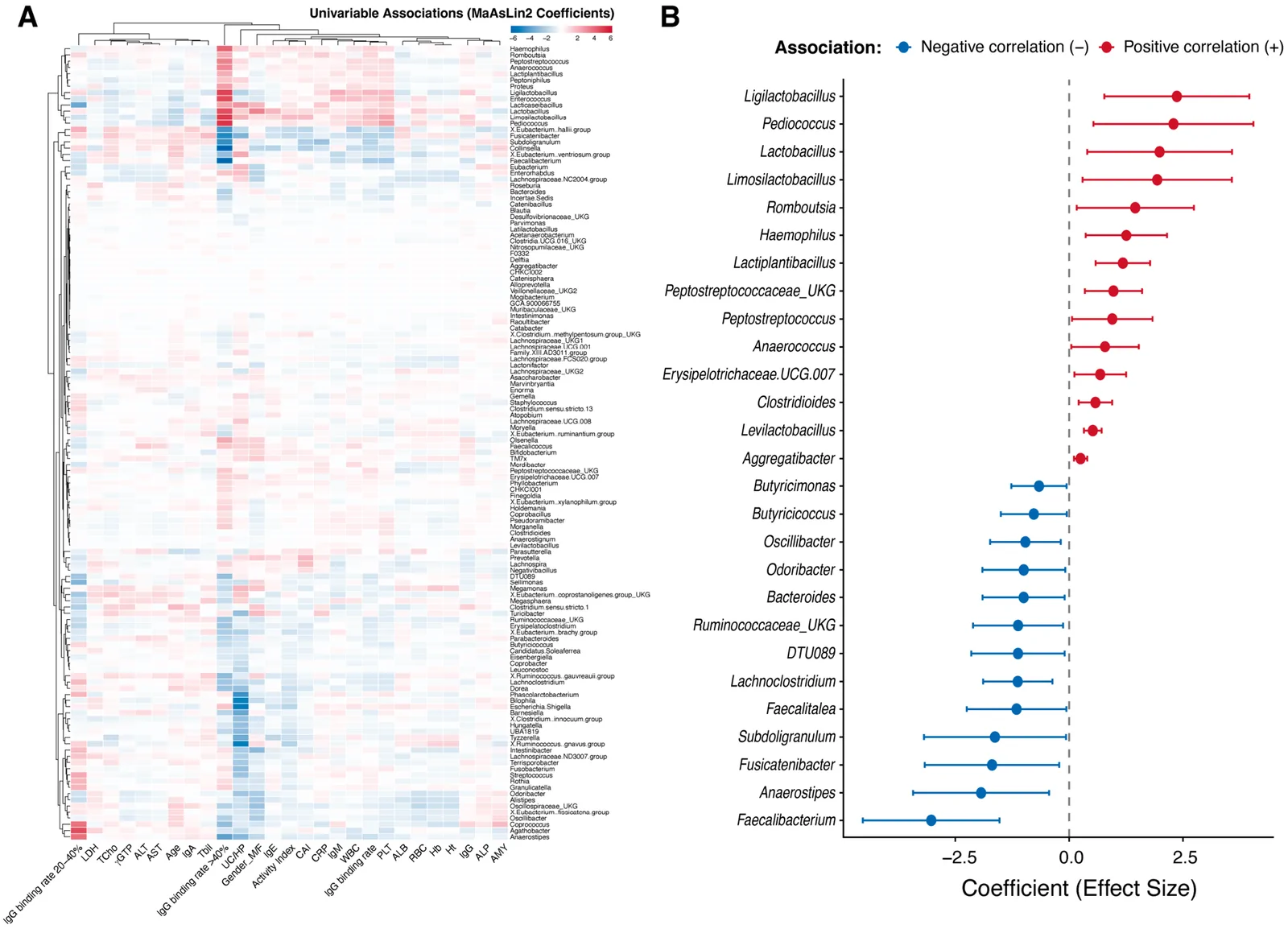
Association analysis of clinical parameters and the IgG binding rate with gut bacterial taxa in the feces fraction. (**A**) Heatmap illustrating the results of univariate association analyses (MaAsLin2) between various clinical parameters and the relative abundance of bacterial genera in the feces fraction. Only bacterial taxa exhibiting a statistically significant association (nominal *p* < 0.05) with at least one clinical variable are included. Cell colors denote the regression coefficients derived from the MaAsLin2 models, with red indicating a positive association and blue indicating a negative association; color intensity reflects the effect size. Rows (bacterial genera) and columns (clinical metadata) are ordered via hierarchical clustering based on the similarity of their abundance profiles. The definitions of the clinical parameters presented in the columns and the reference levels used for categorical contrasts are as follows: Activity index represents an index stratifying disease severity (Healthy = 1, Remission = 2, Active = 3). For categorical contrasts, Gender evaluates the association in males relative to females; IgG binding rate 20–40% and IgG binding rate >40% evaluate associations relative to the <20% reference group among the three IgG binding rate strata (<20%, 20–40%, and >40%); and UC/HP evaluates the association in patients with ulcerative colitis relative to healthy controls. Additionally, IgG binding rate represents the measured total IgG binding rate as a continuous variable. Abbreviations: CAI, Clinical Activity Index; CRP, C-reactive protein; WBC, White blood cell; RBC, Red blood cell; PLT, Platelets; Hb, Hemoglobin; Ht, Hematocrit; ALB, Albumin; Tcho, Total cholesterol; Tbil, Total bilirubin; AST, Aspartate aminotransferase; ALT, Alanine aminotransferase; ALP, Alkaline phosphatase; γGTP, γ-glutamyl transpeptidase; LDH, Lactate dehydrogenase; AMY, Amylase. (**B**) Forest plot illustrating bacterial taxa that exhibited significant associations (nominal *p* < 0.05) with the IgG binding rate in the feces fraction, based on multivariable models adjusted for confounding variables. In this analysis, clinical disease activity (CAI) and patient demographics (age and sex) were incorporated into the models as covariates. Each dot represents a regression coefficient (effect size), and horizontal error bars denote 95% confidence intervals. Red indicates an independent positive association with the IgG binding rate, whereas blue indicates a negative association.

**Figure 4:**
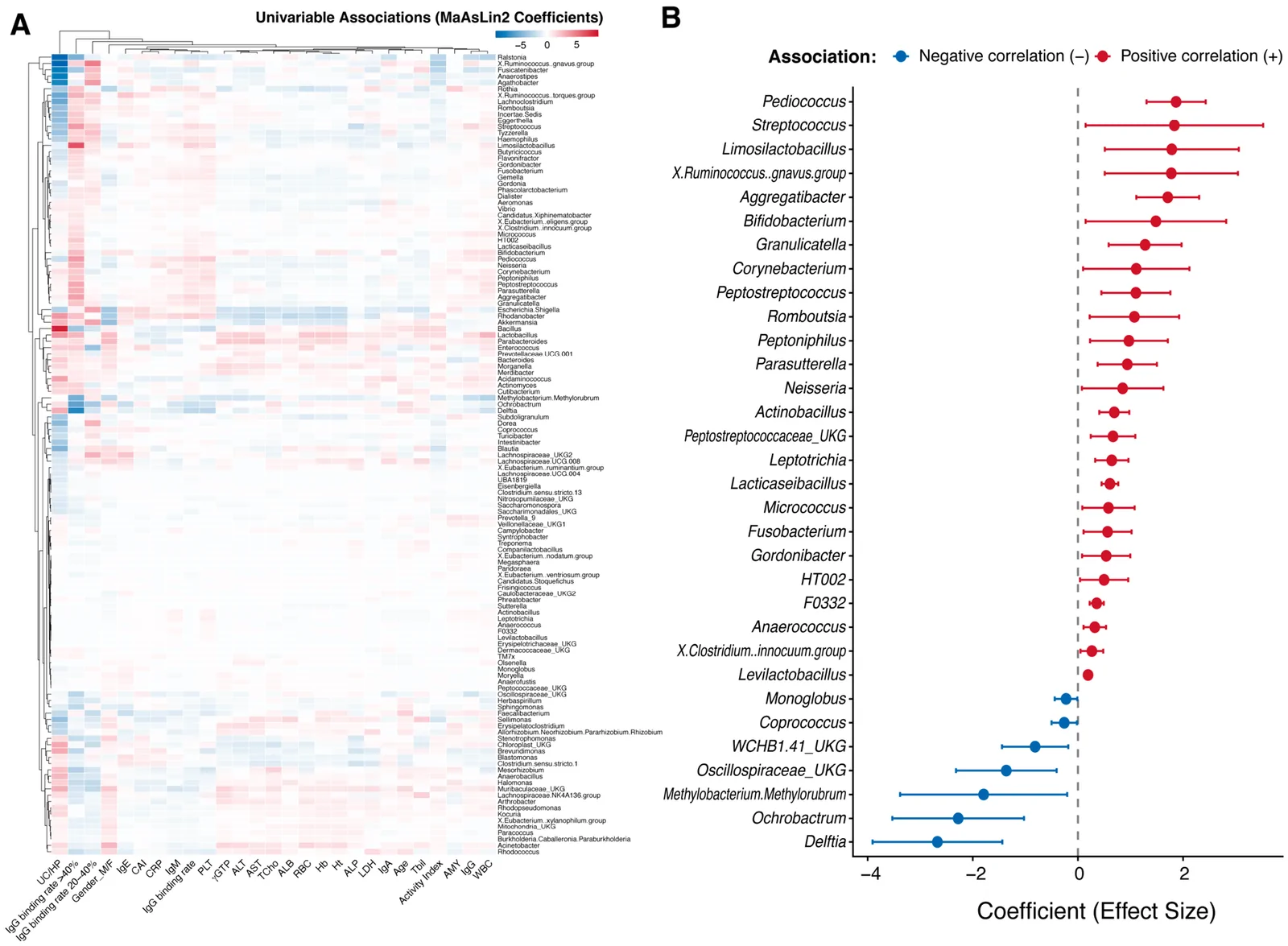
Association analysis of clinical parameters and the IgG binding rate with gut bacterial taxa in the autologous serum IgG-bound (IgG+) fraction. (**A**) Heatmap illustrating the results of univariate association analyses (MaAsLin2) between various clinical parameters and the enrichment of specific bacterial taxa in the IgG-bound fraction. Only bacterial taxa exhibiting a statistically significant association (nominal *p* < 0.05) with at least one clinical variable are included. Cell colors denote the regression coefficients, with red indicating a positive association and blue indicating a negative association. Rows and columns are ordered via hierarchical clustering. The definitions of the clinical parameters presented in the columns and the reference levels used for categorical contrasts are as follows: Activity index represents an index stratifying disease severity (Healthy = 1, Remission = 2, Active = 3). For categorical contrasts, Gender evaluates the association in males relative to females; IgG binding rate 20–40% and IgG binding rate >40% evaluate associations relative to the <20% reference group among the three IgG binding rate strata (<20%, 20–40%, and >40%); and UC/HP evaluates the association in patients with ulcerative colitis relative to healthy controls. Additionally, IgG binding rate represents the measured total IgG binding rate as a continuous variable. Abbreviations: CAI, Clinical Activity Index; CRP, C-reactive protein; WBC, White blood cell; RBC, Red blood cell; PLT, Platelets; Hb, Hemoglobin; Ht, Hematocrit; ALB, Albumin; Tcho, Total cholesterol; Tbil, Total bilirubin; AST, Aspartate aminotransferase; ALT, Alanine aminotransferase; ALP, Alkaline phosphatase; γGTP, γ-glutamyl transpeptidase; LDH, Lactate dehydrogenase; AMY, Amylase. (**B**) Forest plot illustrating bacterial genera that exhibited significant associations (nominal *p* < 0.05) with the IgG binding rate in the IgG-bound fraction, based on multivariable models adjusted for confounding factors. Similar to the analysis of the feces fraction (Figure 3B), the CAI, age, and sex were incorporated as covariates. Each dot represents a regression coefficient (effect size), and horizontal error bars denote 95% confidence intervals. Red indicates an independent positive association with the IgG binding rate, whereas blue indicates a negative association.

To identify bacterial taxa associated with clinical parameters and the autologous serum IgG binding rate, we conducted statistical analyses using MaAsLin2. In this study, we evaluated both the unfractionated whole feces (Feces) and the IgG-bound (IgG+) fractions. Initially, univariate analyses were performed to comprehensively assess the associations between clinical parameters and bacterial taxa (Figure 3A and Figure 4A). In the analysis of the feces fraction (Figure 3A), lactic acid bacteria such as *Ligilactobacillus*, along with *Enterococcus*, exhibited positive associations with the high IgG binding rate group (>40%), whereas taxa such as *Faecalibacterium* and *Subdoligranulum* displayed negative associations. In the IgG+ fraction (Figure 4A), taxa including *Limosilactobacillus* and *Streptococcus*, as well as recognized oral commensals such as *Aggregatibacter*, *Neisseria*, and *Granulicatella*, demonstrated positive associations with the high IgG binding rate group. Furthermore, in both fractions, we identified taxa that correlated not only with disease activity (e.g., CAI, Activity Index) but also with host background factors such as age and sex.

However, the associations identified in these univariate analyses may be subject to confounding by factors such as age, sex, and disease activity. To adjust for these potential confounders, we subsequently conducted multivariable analyses incorporating the CAI, age, and sex as covariates. In the multivariable analysis of the feces fraction, lactic acid bacteria including *Ligilactobacillus*, *Pediococcus*, and *Lactobacillus* exhibited significant positive associations with the IgG binding rate. Conversely, short-chain fatty acid-producing bacteria (SCFA-producing bacteria) such as *Faecalibacterium*, *Anaerostipes*, and *Subdoligranulum* demonstrated negative associations (Figure 3B). Furthermore, multivariable analysis of the IgG+ fraction revealed positive associations between the IgG binding rate and *Pediococcus*, *Limosilactobacillus*, *Bifidobacterium*, the [*Ruminococcus*] *gnavus* group, and oral-associated bacteria such as *Aggregatibacter* (Figure 4B). These results suggest that specific bacterial taxa remain strongly associated with the host humoral immune response, independent of potential confounding by age, sex, and clinical disease activity.

### 2.4. Disease-Associated IgG-Targeted Bacterial Taxa Identified by IgG+ Probability Score (IPS) Profiling

In approaches that isolate and analyze the antibody-bound fraction of the gut microbiota, conventional methods that simply compare relative abundances within the recovered fraction are susceptible to abundance bias. Specifically, bacterial taxa that are highly abundant in the unfractionated whole microbiota (input sample) are frequently overrepresented in the IgG-bound fraction, while low-abundance taxa can cause mathematical inflation when using simple ratio-based indices [33]. To correct for the confounding influence of this baseline abundance and evaluate taxa exhibiting IgG-associated enrichment, we utilized the IgG+ Probability Score (IPS). As described in the methods, this metric incorporates the sample-specific IgG binding rate and an upper-limit clipping method to minimize false positives and compositional artifacts. We subsequently analyzed the associations between the IPS of individual taxa and clinical characteristics.

**Figure 5:**
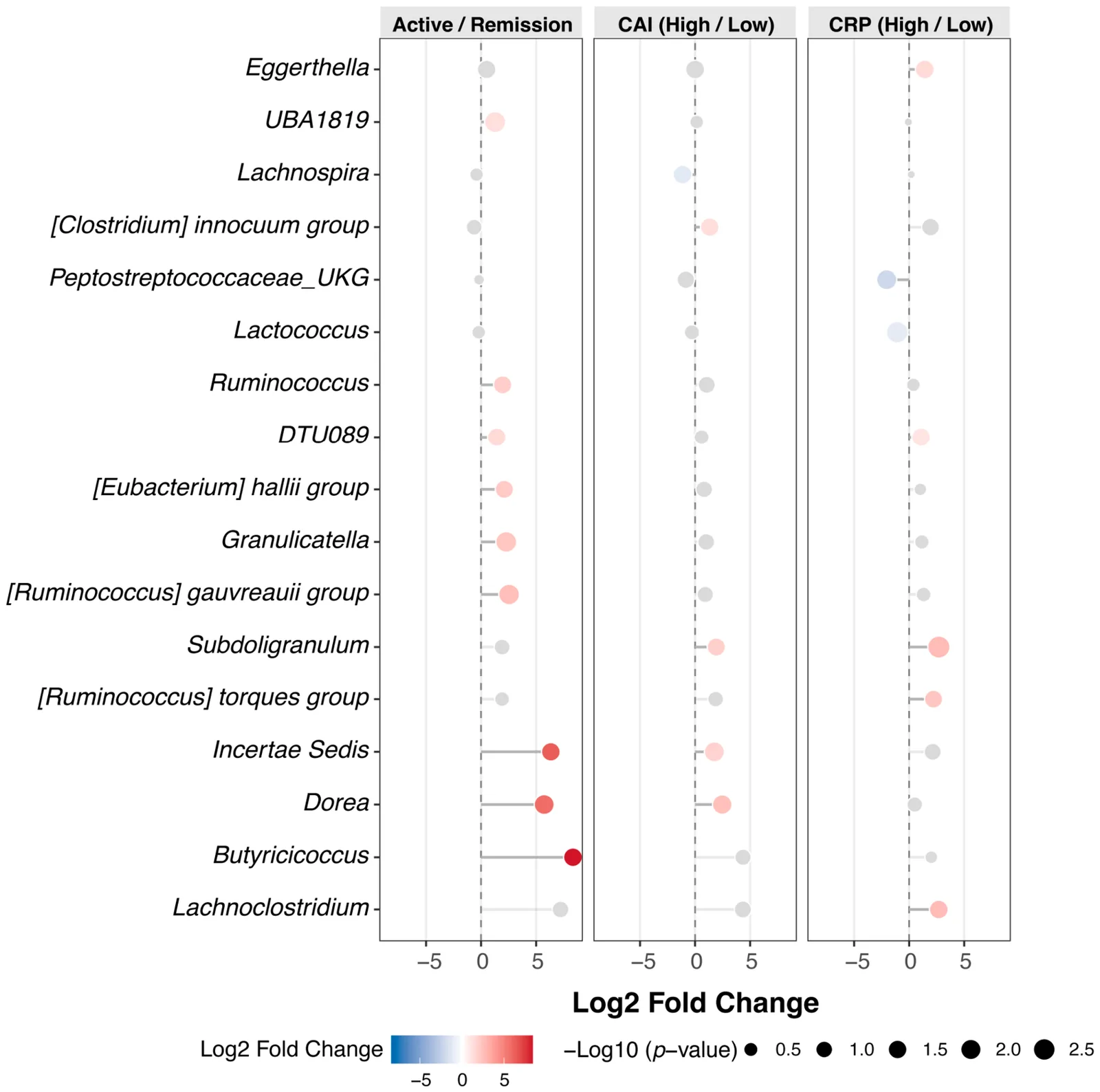
Variations in the IgG+ Probability Score (IPS) of gut bacterial taxa based on the stratification of clinical parameters. Log2 fold changes of the IPS for each bacterial taxon between the two groups in the UC cohort, stratified by clinical parameters. The IPS mathematically integrates the sample-specific IgG binding rate to correct for background bacterial abundance bias, reflecting the relative targeting of each taxon by systemic IgG. On the *x*-axis, a positive log2 fold change (right side) indicates a higher IPS in the corresponding exacerbation or high-value group, whereas a negative value (left side) indicates a higher IPS in the control (remission or low-value) group. The criteria used for the dichotomous stratification of the evaluated clinical parameters are as follows: disease activity was classified into active and remission phases; the Clinical Activity Index (CAI) was divided into high (>3) and low (≤3) groups; and C-reactive protein (CRP) was divided into high (>0.3 mg/dL) and low (≤0.3 mg/dL) groups based on the clinical reference value. Statistical significance between groups was evaluated using the Wilcoxon rank-sum test, with the size of each dot reflecting the level of significance (−log10 nominal *p*-value). Taxa exhibiting statistically significant variations (nominal *p* < 0.05 and |log2 fold change| ≥ 1) are highlighted in colors corresponding to their direction of change, whereas those lacking significant differences under a given condition are shown in gray. Note: Significance thresholds are based on nominal *p*-values. The corresponding Benjamini–Hochberg FDR-adjusted *q*-values are detailed in Table S2.

First, we compared the Log2 Fold Change in the IPS of each bacterial taxon between dichotomized clinical parameter groups (e.g., active phase vs. remission, high vs. low CAI, high vs. low CRP) (Figure 5). The analysis revealed that during the active phase of the disease and in groups with high CAI (an indicator of clinical disease activity), the IPS was significantly elevated for taxa belonging to the family Lachnospiraceae (e.g., the [*Ruminococcus*] *gauvreauii* group and *Dorea*) as well as the family Ruminococcaceae (e.g., *Ruminococcus*, *UBA1819*, *Incertae Sedis*, and *DTU089*). Recognized oral commensals such as *Granulicatella*, along with the [*Eubacterium*] *hallii* group, also showed elevated IPS in these active groups. Interestingly, *Butyricicoccus*, which is generally considered to contribute to the maintenance of intestinal homeostasis, also exhibited an increased IPS during the active phase. Furthermore, in patients with elevated CRP levels (a systemic inflammatory marker), there was an increase in the IPS of taxa including the butyrate-producing bacterium *Subdoligranulum* and mucin-degrading taxa such as *Lachnoclostridium* and the [*Ruminococcus*] *torques* group. Conversely, in groups with low inflammatory markers (low CAI and low CRP), taxa such as *Lactococcus*, *Lachnospira*, and *Peptostreptococcaceae*_UKG demonstrated a tendency to be relatively enriched as IgG targets. These results suggest that the profile of bacterial populations specifically targeted by host IgG responses differs in accordance with the disease phase and systemic inflammatory status. However, it should be noted that the enrichment of these specific taxa (e.g., *Granulicatella*, *Subdoligranulum*) is based on nominal statistical significance (nominal *p* < 0.05). These differences did not remain statistically significant after adjusting for multiple comparisons using the Benjamini–Hochberg FDR correction. Full statistical results, including nominal *p*-values and adjusted *q*-values for all screened taxa, are provided in Table S2.

**Figure 6:**
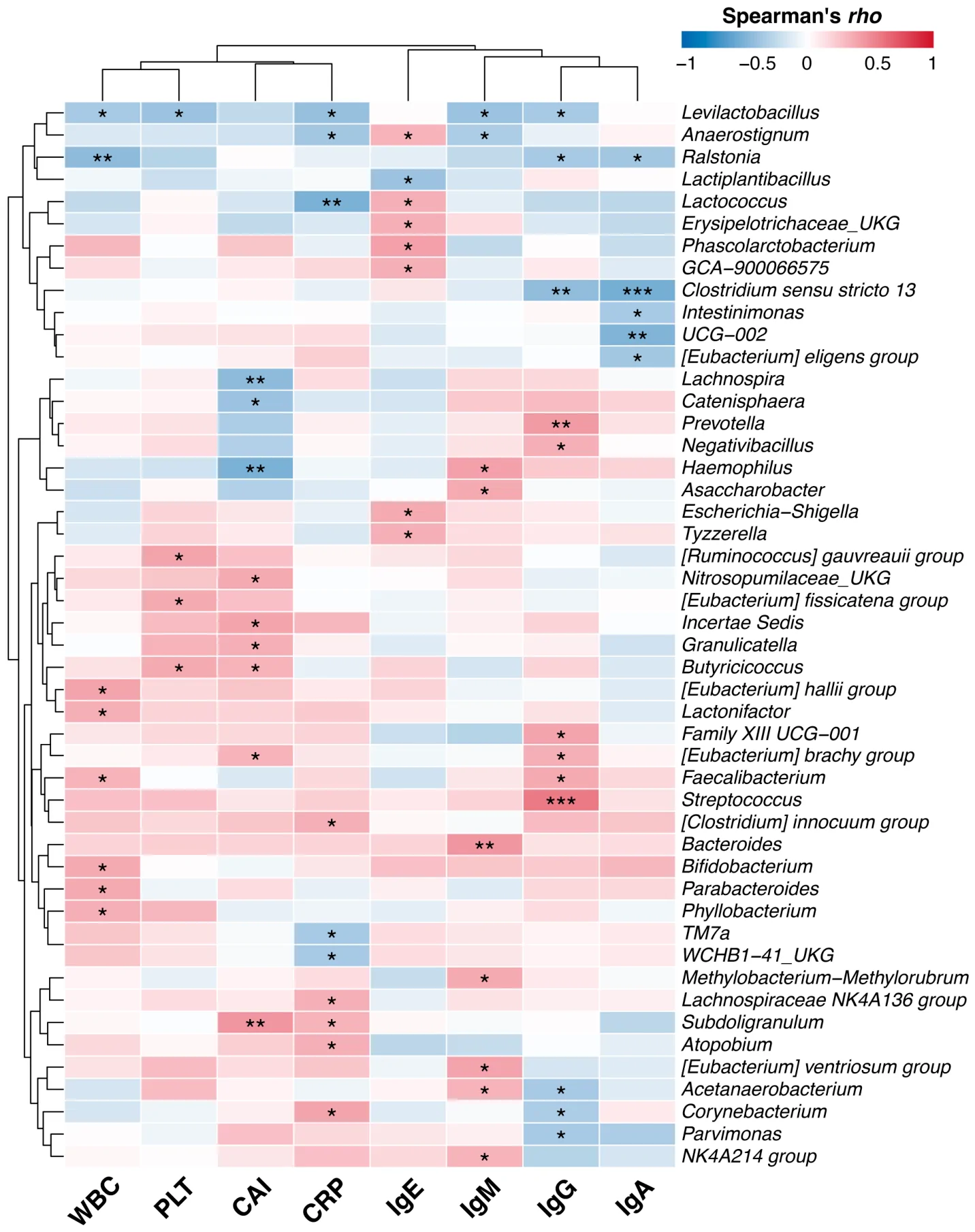
Correlation analysis between the IgG+ Probability Score (IPS) of bacterial taxa and continuous clinical parameters. Heatmap illustrating the Spearman’s rank correlation coefficients (Rho) between the IPS of individual bacterial taxa and various clinical parameters evaluated as continuous variables (WBC, PLT, CAI, CRP, IgE, IgM, IgG, and IgA) in patients with UC. Following a comprehensive pairwise correlation analysis across all taxa, the plot displays the bacterial taxa exhibiting the strongest significant correlations with at least one clinical parameter. Cell colors indicate the direction and magnitude of the correlation coefficient (Rho), with red representing a positive correlation and blue representing a negative correlation. Rows (bacterial taxa) and columns (clinical metadata) are ordered via hierarchical clustering based on the similarity of their correlation patterns. Asterisks within the cells denote the levels of nominal statistical significance for the correlations (* *p* < 0.05, ** *p* < 0.01, *** *p* < 0.001). The corresponding Benjamini–Hochberg FDR-adjusted *q*-values for all correlations are detailed in Table S3. Abbreviations: CAI, Clinical Activity Index; CRP, C-reactive protein; WBC, White blood cell; PLT, Platelets.

To further evaluate the direct continuous associations between the IPS of individual taxa and clinical metadata, we performed Spearman’s rank correlation analysis and generated a clinical correlation heatmap (Figure 6). This analysis identified *Subdoligranulum*, *Incertae Sedis*, *Granulicatella*, and *Butyricicoccus* as taxa exhibiting significant positive correlations with the CAI. Moreover, the IPS of the [*Clostridium*] *innocuum* group and *Subdoligranulum* demonstrated significant positive correlations with CRP. Additionally, taxa such as *Butyricicoccus*, the [*Eubacterium*] *fissicatena* group, and the [*Ruminococcus*] *gauvreauii* group exhibited significant positive correlations with platelet (PLT) counts, another crucial marker of systemic inflammation. Notably, lactic acid bacteria such as *Levilactobacillus*, which exhibited increased relative abundances in both the feces and IgG+ fractions in the earlier multivariable analyses (Figure 3 and Figure 4), demonstrated negative correlations with the CAI, CRP, and PLT when evaluated using the IPS. This discrepancy underscores that while these taxa expand in relative abundance within the inflammatory milieu, they are not the primary, specific targets of the systemic IgG response. These findings suggest that integrating standard abundance profiling with the IPS metric identifies bacterial populations whose IgG-targeting patterns are associated with markers of clinical disease activity and systemic inflammation in UC.

To validate whether the observed associations between the IPS and clinical parameters reflect true taxon-specific IgG enrichment rather than being confounded by the overall IgG-binding rate, we conducted a sensitivity analysis. We calculated an alternative metric, the IgG Coating Index (ICI), defined as a simple ratio of relative abundance in the IgG+ fraction to that in the whole feces, which does not incorporate the sample-specific overall IgG-binding rate (Figure S3A). This analysis confirmed that key taxa, including *Granulicatella*, *Subdoligranulum*, *Dorea*, and the *[Ruminococcus] torques* group, remained enriched in the active phase, high CAI and high CRP groups. Furthermore, the simple ICI metric identified additional taxa, such as *Neisseria* and *Actinobacillus*, as associated with elevated CRP, which were not extracted by the IPS. As demonstrated in Figure S3B, because the ICI relies on a simple ratio, it is mathematically susceptible to extreme artificial inflation when a taxon’s baseline abundance is near zero. These findings demonstrate that the observed associations are not solely attributable to the incorporation of the overall IgG-binding rate, and that the IPS calculation filters out mathematical artifacts associated with low-abundance taxa.

### 2.5. Functional Characteristics of IgG-Targeted Bacterial Taxa and Their Association with Disease Activity

**Figure 7:**
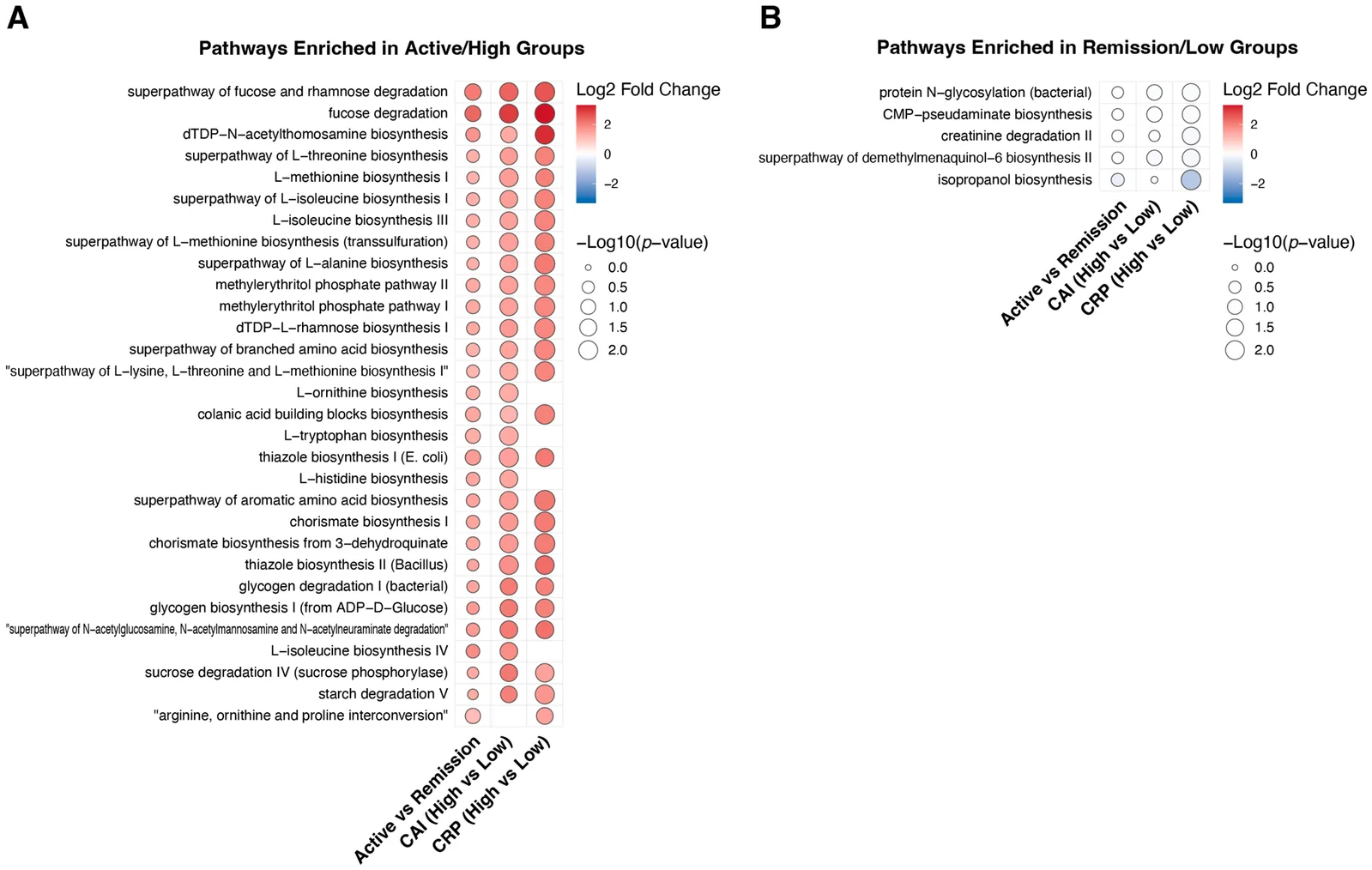
Variation profile of the functional IgG+ Probability Score (IPS) in predicted metabolic pathways based on clinical parameters. Bubble heatmap illustrating variations in the functional IPS of predicted metabolic pathways across dichotomous comparisons for each clinical phenotype. Metabolic pathway profiles were predicted using PICRUSt2 based on 16S rRNA gene sequencing data. The evaluated clinical parameters comprise disease activity (active vs. remission phase), as well as the Clinical Activity Index (CAI) and C-reactive protein (CRP) levels (high vs. low groups for each). The functional IPS mathematically integrates the sample-specific IgG binding rate to correct for background abundance bias, reflecting the relative targeting of each predicted metabolic pathway by systemic IgG. Among the pathways exhibiting statistically significant variations (nominal *p* < 0.05, Wilcoxon rank-sum test) in each two-group comparison, the top 30 pathways with the highest absolute log2 fold changes were extracted and plotted for each direction of variation. Pathways on the *y*-axis are ordered via hierarchical clustering based on the similarity of their variation patterns. The color of the dot in each cell represents the log2 fold change, with red indicating a relative increase (upregulation) of the functional IPS in the exacerbated groups (active phase or high-value groups) and blue indicating a relative increase in the control groups (remission phase or low-value groups). The size of each dot reflects the magnitude of statistical significance (−log10 nominal *p*-value) for each comparison. Note: The visualization is based on nominal *p*-values. The corresponding Benjamini–Hochberg FDR-adjusted *q*-values are detailed in Table S4. Abbreviations: CAI, Clinical Activity Index; CRP, C-reactive protein. (**A**) The top 30 metabolic pathways exhibiting higher predicted abundances in the clinically exacerbated and high immune response groups. (**B**) The top 30 metabolic pathways exhibiting higher predicted abundances in the remission and low immune response groups (only five pathways met the nominal significance and fold-change criteria).

To investigate the functional potential of the candidate IgG-targeted bacterial taxa, we conducted predictive metabolic pathway profiling using PICRUSt2. We compared the functional IPS of metabolic pathways between dichotomized clinical parameter groups (e.g., active phase vs. remission, high vs. low CAI, high vs. low CRP) (Figure 7A,B). The analysis revealed that groups with high disease activity and elevated inflammatory markers (active, high CAI, and high CRP) exhibited higher predicted abundances of pathways associated with the degradation of host-derived glycoproteins (nominal *p* < 0.05, Figure 7A and Table S4). Specifically, the superpathway of fucose and rhamnose degradation, fucose degradation, and the superpathway of N-acetylglucosamine, N-acetylmannosamine, and N-acetylneuraminate degradation were enriched. After adjusting for multiple comparisons using the Benjamini–Hochberg FDR correction, several pathways (including the superpathway of N-acetylglucosamine, N-acetylmannosamine, and N-acetylneuraminate degradation) remained statistically significant (*q* < 0.05) in the high CRP group. Conversely, because functional profiles in the active and high CAI groups did not pass the stringent FDR correction threshold, those specific comparisons are presented as exploratory signals. Given that fucose and N-acetylneuraminate (sialic acid) are core components of intestinal mucins, this functional profile strongly aligns with the IgG-targeting of mucin-degrading taxa observed during the active phase of the disease. Furthermore, a broad array of *de novo* amino acid biosynthesis pathways was prominently predicted to be enriched in the inflammatory state, specifically those for L-threonine, L-methionine, L-isoleucine, branched-chain amino acids, and aromatic amino acids. Additionally, pathways related to cell surface polysaccharide and capsular biosynthesis, such as dTDP-L-rhamnose biosynthesis I and colanic acid building blocks biosynthesis, showed higher predicted abundances, suggesting a hypothetical adaptation to environmental stress and immune pressure within the inflammatory milieu.

Conversely, in the remission phase and low-index groups, a constrained set of metabolic pathways was enriched as IgG targets (Figure 7B). These included bacterial protein N-glycosylation, CMP-pseudaminate biosynthesis, creatinine degradation II, and the superpathway of demethylmenaquinol-6 biosynthesis II. The enrichment of these predicted pathways may indicate differences in the metabolic potential of IgG-targeted communities under less inflammatory conditions. These findings demonstrate that the predicted functional profiles of IgG-targeted bacterial populations are associated with disease activity and systemic inflammation, specifically highlighting a predicted functional potential for host mucin degradation and extensive amino acid synthesis during UC exacerbation.

### 2.6. Construction of UC Disease Exploratory Classification Models Based on IPS Profiles and Identification of Key Bacterial Taxa

**Figure 8:**
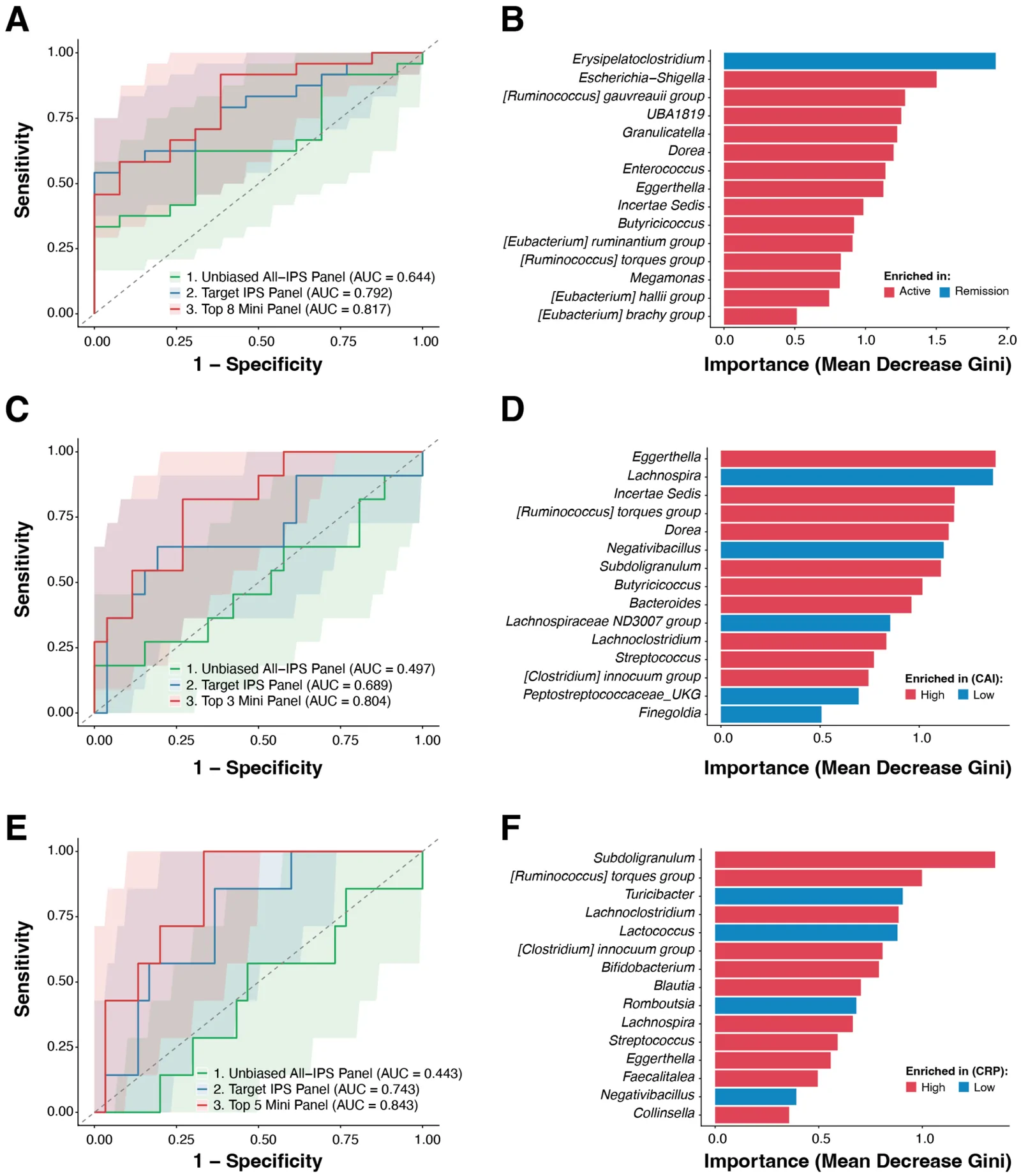
Exploratory classification modeling of ulcerative colitis clinical outcomes and identification of key taxa based on IgG+ Probability Score (IPS) profiling. (**A**,**C**,**E**) ROC curves of classification models for three distinct clinical outcomes, constructed using the Random Forest algorithm. The classification targets comprise disease exacerbation (Active vs. Remission) in (**A**), clinical disease activity (high vs. low CAI) in (**C**), and systemic inflammation (high vs. low CRP) in (**E**). Each plot compares the predictive accuracy (AUC) among three models: a model incorporating the IPS of all bacterial taxa as input variables (1. Unbiased All-IPS Panel, green); a model restricted to pre-selected taxa (2. Target IPS Panel, blue); and an optimized model reconstructed using only the top 3 to 8 taxa extracted from the Target IPS Panel based on variable importance (3. Top Mini Panel, red). The shaded regions surrounding each ROC curve denote the 95% confidence intervals for sensitivity, calculated via bootstrapping. (**B**,**D**,**F**) Bar plots illustrating the top-ranking taxa based on variable importance (Mean Decrease Gini) within the Target IPS Panel models for each prediction target. Plot (**B**) corresponds to the classification model for disease exacerbation, (**D**) for CAI, and (**F**) for CRP. The color of each bar indicates the directional clinical phenotype in which the IPS of the respective taxon was relatively enriched in prior analyses: red reflects specific enrichment in the exacerbated groups (active phase or high-value groups), whereas blue reflects specific enrichment in the control groups (remission phase or low-value groups). Abbreviations: CAI, Clinical Activity Index; CRP, C-reactive protein. Note: AUC values are within-cohort (apparent) estimates. Feature selection (Target IPS Panel), determination of the number of taxa (Top Mini Panel), and ROC evaluation were all performed on the same dataset without nested cross-validation or an independent validation cohort; the values are therefore likely to be optimistic and should not be interpreted as validated predictive or diagnostic performance.

To evaluate the potential utility of IgG-targeted bacterial profiles in differentiating clinical phenotypes, machine learning models were constructed using the Random Forest algorithm, and exploratory discriminatory capacity was assessed by receiver operating characteristic (ROC) analysis. Three clinical outcomes were used as classification targets: UC exacerbation (active vs. remission), clinical disease activity (CAI), and systemic inflammation (CRP) (Figure 8). It should be emphasized that all AUC values reported in this section are within-cohort (apparent) estimates. Because feature selection, determination of the number of predictors, and performance evaluation were all performed on the same dataset without nested cross-validation or an independent validation cohort, these values are likely to overestimate performance in independent samples and should be interpreted as exploratory. We compared model performances across a stepwise feature-selection strategy. An unbiased model incorporating the IPS of all bacterial taxa (1. Unbiased All-IPS Panel) exhibited limited discriminatory capacity, yielding area under the curve (AUC) values of 0.644 for exacerbation (Figure 8A), 0.497 for CAI (Figure 8C), and 0.443 for CRP (Figure 8E). To improve discriminatory capacity, we refined the features to a Target IPS Panel comprising the top 15 taxa identified by variable importance in the initial unbiased models. This refinement was accompanied by higher apparent AUC values (0.792, 0.689, and 0.743, respectively), although this increase may partly reflect selection bias. Furthermore, iterative feature selection generated compact candidate panels (3. Top Mini Panel) requiring only 3 to 8 candidate taxa (Figure S4). These optimized panels yielded the highest apparent AUCs within this cohort, with AUCs of 0.817 for exacerbation (Top 8 Mini Panel), 0.804 for CAI (Top 3 Mini Panel), and 0.843 for CRP (Top 5 Mini Panel).

Analysis of variable importance (Mean Decrease in Gini impurity) within the Target IPS Panels revealed distinct profiles of predictive taxa depending on the clinical target (Figure 8B,D,F). In the classification model for UC exacerbation (Figure 8B), taxa such as *Erysipelatoclostridium*, *Escherichia-Shigella*, and the [*Ruminococcus*] *gauvreauii* group demonstrated the highest contributions. In the classification model for the CAI (Figure 8D), *Eggerthella*, *Lachnospira*, and *Incertae Sedis* emerged as the top-ranking important variables. For the classification of systemic inflammation represented by CRP levels (Figure 8F), *Subdoligranulum* was identified as the strongest predictor, followed by the [*Ruminococcus*] *torques* group and *Turicibacter*. These findings suggest that the IPS of a limited set of candidate IgG-targeted taxa, selected according to distinct disease dimensions such as clinical disease activity or systemic inflammation, may capture disease-related signals that differ among clinical outcomes. Because the performance estimates are non-validated and likely optimistic, this analysis should be regarded as a means of prioritizing candidate taxa for future validation rather than as evidence of superior classification performance.

## 3. Discussion

In the present study, we investigated candidate bacterial taxa targeted by systemic IgG responses and their predicted metabolic pathways using fecal samples and autologous sera from patients with UC. Our results revealed that the relative abundance of gut bacteria did not necessarily align with their probability of being bound by IgG, indicating that systemic humoral immune responses are selectively directed toward specific bacterial taxa. Indeed, lactic acid bacteria such as *Levilactobacillus* and *Lactiplantibacillus*, which demonstrated significant increases in relative abundance within both the unfractionated feces and IgG-bound fractions (Figure 3 and Figure 4), exhibited negative correlations with systemic inflammatory markers when evaluated by the IgG+ Probability Score (IPS) (Figure 5 and Figure 6). Conversely, we identified distinct taxa that exhibited a high IPS despite not being highly abundant in the overall microbial community. These findings are consistent with previous reports demonstrating that IgG responses in patients with IBD are not uniformly directed across the entire gut microbiota, but rather selectively target specific bacterial taxa [31,32,33]. Furthermore, the conventional evaluation of IgG-bound fractions is inherently susceptible to abundance bias driven by the background population [33]. In our sensitivity analysis utilizing a simple ratio-based metric without the global IgG-binding rate (the ICI), we observed consistent enrichment of key taxa such as *Granulicatella* and *Subdoligranulum*, demonstrating that these associations are not solely attributable to the incorporation of the overall IgG-binding rate into the IPS. However, the simple ratio calculation also demonstrated severe mathematical inflation for ultra-low abundance taxa. By integrating the sample-specific IgG binding rate and an upper-limit clipping method, the IPS utilized in this study mitigates the mathematical inflation of simple ratio-based metrics and the confounding influence of baseline abundance, providing an exploratory method for identifying candidate bacterial populations whose relative enrichment is consistent with systemic humoral immunity.

The experimental system employed in this study was designed to evaluate bacterial taxa targeted by systemic IgG responses through an in vitro reaction between patient-derived fecal bacteria and autologous serum. Previous studies have demonstrated that the systemic antimicrobial IgG repertoire reflects bacterial taxa that have translocated across the intestinal barrier into host tissues [32]. Accordingly, profiling serum IgG responses serves as an approach for identifying bacterial taxa immunologically exposed to the host. In the present study, an increased IPS against recognized oral commensals was observed; specifically, the IPS of *Granulicatella* was elevated in patients with active UC and high CAI, while *Atopobium* and *Corynebacterium* demonstrated significant positive correlations with CRP (Figure 6). Previous studies have hypothesized that such bacterial populations may induce systemic IgG responses through ectopic colonization of the intestinal tract and increased immune exposure resulting from impaired mucosal barrier function. However, because our study analyzed fecal samples using 16S rRNA gene sequencing without paired oral microbiome sampling, mucosal biopsies, or actual in vivo measurements of tissue translocation, our data do not directly demonstrate oral-to-gut transmission or ectopic colonization. Rather, our findings demonstrate an association between systemic IgG targeting and taxa commonly found in the oral microbiota. Similar observations have been reported by Rengarajan et al. and Bourgonje et al., who demonstrated that systemic and local IgG responses in patients with IBD are not uniformly directed toward the entire gut microbiota but are preferentially targeted toward specific bacterial taxa [28,31]. In their studies, IgG responses against dominant anaerobic commensals in the colon, such as *Faecalibacterium* and *Roseburia*, were relatively limited, whereas robust IgG binding was observed against oral-associated taxa, including *Veillonella*, *Gemella*, and *Peptostreptococcus*. These insights suggest that alterations in microbial community structure, together with the intestinal colonization of bacteria outside their native ecological niches, contribute to changes in host humoral immune responses. Recently, oral- and upper gastrointestinal tract-derived bacteria have been reported to become enriched within the mucosa-associated microbiota (MAM) of pediatric UC patients in parallel with increasing disease activity and mucosal inflammation [37]. The influx and colonization of oral bacteria in the gut have been implicated in IBD pathogenesis under the concept of the oral–gut axis [11,13,20,21,22]. In this context, the elevated systemic IgG targeting of oral-associated taxa observed in our study suggests a potential link between these taxa and the elicitation of systemic humoral immunity, although providing direct evidence of their origin and migration mechanisms requires further investigation.

Impaired mucosal barrier function is considered a key factor facilitating bacterial translocation into host tissues. In this study, mucin-degrading taxa such as *Lachnoclostridium* and the [*Ruminococcus*] *torques* group, along with specific members of the family Lachnospiraceae (e.g., *Dorea* and the [*Ruminococcus*] *gauvreauii* group), exhibited significantly elevated IPS in the active UC, high CAI, and high CRP groups (Figure 5). These specific taxa are known to possess mucin-foraging capabilities, enabling them to utilize host-derived mucins as a nutrient source [38,39,40]. Recent studies have demonstrated that the thinning of the mucus layer and alterations in the mucus environment influence host–microbe interactions by increasing epithelial permeability and promoting bacterial contact with the mucosal surface [41]. Desai et al. showed that the expansion of mucin-utilizing bacteria under conditions of dietary fiber deficiency leads to the erosion of the mucus barrier and facilitates closer bacterial proximity to the intestinal epithelium [41]. However, the present study did not directly evaluate mucus thickness, epithelial permeability, bacterial localization, or in vivo mucin degradation activity. Furthermore, functional characteristics may differ at the species or strain level, which cannot be fully resolved by 16S rRNA gene sequencing. Therefore, rather than providing direct evidence that mucosal barrier disruption or actual mucin degradation is occurring through these specific bacteria, the enhanced IgG responses observed in our study suggest a possible link between the IgG recognition of putative mucin-utilizing taxa and the host inflammatory status.

It is also crucial to note that systemic IgG responses against the gut microbiota reflect a complex interplay between immune defense and mucosal homeostasis. Recent large-scale metagenomic analyses have reported that *Eggerthella lenta* encompasses not only disease-adapted strains that proliferate in the inflammatory environment of IBD but also health-associated strains that inversely correlate with disease severity [42]. In the present study, *Eggerthella* exhibited an elevated IPS in the high CRP group. This finding suggests that these elevated IgG responses may preferentially reflect lineages associated with the inflammatory environment, although strain-level resolution was not available to definitively confirm this. Conversely, systemic IgG targeting does not exclusively signify disease exacerbation. In the remission phase and low inflammatory marker groups, distinct taxa such as *Lactococcus* and *Lachnospira* demonstrated a tendency to be relatively enriched as IgG targets. Systemic IgG and intestinal secretory IgA are known to share certain commensal bacteria as common targets; given that anti-commensal IgG responses are augmented in selective IgA deficiency, it has been proposed that IgG functions as a compensatory defense mechanism complementing mucosal immunity [43]. In addition, autologous serum IgG derived from patients with UC exhibits high reactivity toward specific commensals, including Lactobacillaceae (e.g., *Lacticaseibacillus paracasei*) [44]. IgG binding to these bacteria has been suggested to influence complement activation associated with immune complex formation involving other species (e.g., *Escherichia coli*) [44]. Taken together, these findings suggest that systemic IgG responses are not solely responsible for neutralizing pathogenic bacteria but are also actively involved in maintaining homeostatic interactions with the gut microbiota. Therefore, the specific IgG-targeted taxa identified in this study reflect a dynamic, adaptive host immune response shifting between inflammatory defense and immune homeostasis. Because the absolute pathogenic or protective roles of individual bacterial taxa cannot be directly determined solely from the IgG binding patterns observed herein, future studies incorporating strain-level analyses and mechanistic investigations are warranted to further elucidate the precise roles of these IgG-targeted populations in UC pathogenesis.

The observed association between the IgG response against the genus *Subdoligranulum* and systemic inflammatory markers (CRP) aligns with previous studies demonstrating a relationship between this taxon and host immune responses [45,46]. *Subdoligranulum* is generally recognized as a butyrate-producing bacterium that contributes to the maintenance of intestinal homeostasis [47], but certain lineages have been reported to exhibit cross-reactivity with autoantibodies and have been implicated in autoimmune pathogenesis [46]. Furthermore, our analysis revealed that *Butyricicoccus*, another critical butyrate-producing commensal, exhibited an increased IPS during the active phase and significantly correlated with the CAI and platelet counts. *Butyricicoccus* is a butyrate-producing genus that has been reported to enhance intestinal epithelial barrier integrity and is consistently depleted in patients with IBD, making it a promising next-generation probiotic candidate [48,49]. Despite their well-established protective roles, the increased IgG targeting of both *Subdoligranulum* and *Butyricicoccus* in highly active and inflammatory states may reflect an altered relationship in mucosal immune tolerance. One possible explanation is that disruption of the mucosal barrier during UC exacerbation increases the exposure of these mucosa-associated, beneficial commensals to the systemic immune system, resulting in enhanced systemic IgG recognition. These findings suggest that systemic IgG responses in active UC are not restricted to putative pathobionts but may also target commensal bacteria with established beneficial functions.

Consistent with these taxonomic shifts, our predictive functional profiling corroborated a predicted enrichment of metabolic pathways associated with the degradation of host-derived mucin glycoproteins (e.g., fucose and sialic acid) during UC exacerbation (Figure 7A). The predicted functional enrichment of these specific pathways directly aligns with the elevated systemic IgG targeting of mucin-degrading taxa observed in our IPS analysis. Additionally, pathways involved in cell surface polysaccharide and capsular biosynthesis, including colanic acid building blocks and dTDP-L-rhamnose biosynthesis, showed higher predicted abundances. Capsular polysaccharides are well-recognized virulence determinants that protect bacteria from host immune defenses, including phagocytosis and complement-mediated killing, while enhancing survival under inflammatory conditions. The predicted enrichment of these pathways leads to the hypothesis that it may therefore reflect adaptive remodeling of bacterial surface structures in response to the inflammatory and oxidative microenvironments [50,51,52]. Furthermore, multiple *de novo* amino acid biosynthesis pathways showed higher predicted abundances in the IgG-targeted microbiota of patients with highly active disease. We hypothesize that this observation may reflect metabolic adaptation of these bacterial populations to the altered nutrient environment associated with intestinal inflammation, which is known to profoundly reshape microbial metabolic activity in IBD [53,54]. However, it must be acknowledged that this analysis relies on functional predictions derived from 16S rRNA gene sequencing data. It does not measure gene expression, pathway activity, metabolite production, or actual bacterial adaptation. Consequently, these mechanistic interpretations remain hypotheses, and further validation utilizing shotgun metagenomic, metatranscriptomic, and metabolomic analyses is warranted to definitively confirm the functional characteristics of the IgG-targeted microbial populations.

In the machine learning analysis, the profiles of the candidate IgG-targeted bacterial taxa contributing to the classification of UC exacerbation (active vs. remission), clinical disease activity (CAI), and systemic inflammation (CRP) differed. This indicates that the bacterial populations targeted by systemic IgG responses may reflect different facets of UC pathogenesis, encompassing both clinical disease activity and systemic immunological status. The higher apparent AUCs of the optimized models (Top Mini Panels) relative to the Unbiased All-IPS Panel must not be interpreted as evidence of improved classification performance. Because the top 15 taxa, the number of predictors in the Mini Panels, and the final AUC were all determined using the same cohort without nested cross-validation or an independent validation cohort, these values likely reflect information leakage and overfitting and are expected to overestimate performance in independent samples. In a cohort of this size (*n* = 37 patients with UC), such optimism may be substantial. Accordingly, the AUC values reported here should be regarded as descriptive, within-cohort summaries rather than validated predictive performance. Therefore, the primary significance of this analysis lies not in the classification metrics themselves, but rather in the exploratory identification of candidate IgG-targeted taxa associated with different clinical parameters, underscoring the potential of these immune–microbe interactions to mirror the multi-faceted clinical characteristics of UC. As this study is an exploratory analysis conducted on a relatively small cohort, future studies utilizing independent validation cohorts are essential to rigorously evaluate whether the candidate taxa identified herein are generalizable to broader clinical populations.

Several limitations of this study should be acknowledged. First, while the IPS introduced herein mathematically integrates the sample-specific IgG-binding rate to mitigate background abundance bias, the underlying 16S rRNA gene sequencing data remains compositional [55]. Consequently, the IPS should not be interpreted as an absolute quantitative measure of IgG mass bound to each taxon, but rather as a probabilistic index reflecting the relative targeting of bacterial taxa by systemic IgG. Second, our experimental design utilized an in vitro assay reacting patient-derived fecal microbiota with autologous serum. Thus, the identified taxa represent bacteria with the capacity to be targeted by systemic IgG, which does not directly confirm their actual in vivo state of antibody coating or tissue translocation within the intestinal tract at the time of sampling. Furthermore, a mock control (e.g., bead-only or no-IgG) was not included during the magnetic recovery process. Consequently, the possibility of non-specific bacterial adherence to the Protein G beads cannot be entirely excluded. Third, the functional profiling was predictive, relying on PICRUSt2, and does not directly evaluate true gene expression or metabolic activity. Moreover, recent studies have shown that antibody-targeting patterns can vary substantially not only at the species level but also at the strain level [35]. However, the taxonomic resolution of the present study was insufficient to evaluate such strain-level differences. Future validation using shotgun metagenomic and metatranscriptomic approaches will therefore be required. Fourth, secretory IgA was not evaluated in this study, and thus the relationship between mucosal immune responses and systemic IgG responses remains unclear [27,28,43]. Fifth, the very small size of the healthy control group (*n* = 4) is a major limitation, as it restricts our ability to adequately capture the natural inter-individual variability of the healthy gut microbiota and systemic IgG responses. Therefore, all comparisons between UC patients and healthy participants in this study are exploratory. Further studies with larger, well-matched control cohorts are necessary to confirm whether the observed microbial signatures are characteristic of UC relative to the healthy population. Sixth, although our multivariable models adjusted for age, sex, and the CAI, we did not match or adjust for other microbiome-related confounding factors. While the pharmacological treatments in our cohort were limited to 5-aminosalicylic acid and probiotics (with no concurrent use of corticosteroids, immunomodulators, biologics, or antibiotics), we could not incorporate all clinical variables, such as the specific probiotic strains used, disease duration, and diet, as covariates into the statistical models due to the limited cohort size. Therefore, the potential for substantial residual confounding cannot be excluded. Seventh, disease activity in this study was evaluated exclusively using the CAI. Because we did not include endoscopic scores (e.g., the Mayo Endoscopic Subscore), which are essential for objectively confirming mucosal healing, the robustness of the clinical correlations and the interpretation of disease severity may be limited. Eighth, a critical statistical limitation is that while we identified several targeted taxa and functional pathways showing nominal statistical significance (*p* < 0.05), many of these features did not survive stringent FDR correction for multiple testing. This loss of significance, particularly in the disease activity and CAI comparisons, is likely attributable to the limited statistical power inherent in our small cohort size combined with the high-dimensional nature of the microbiome data. Therefore, biological interpretations based on features that only achieved nominal significance must be regarded as exploratory signals that require validation in larger, independent cohorts. Finally, it is critical to explicitly distinguish between statistical association, immune recognition, and biological causation. Because this was a relatively small cross-sectional cohort study, it is not possible to determine whether the observed IgG targeting actively contributes to disease pathogenesis or arises merely as a consequence of the inflammatory environment. Furthermore, the specific antigenic molecules recognized by these IgG antibodies remain unidentified. Future longitudinal cohort studies, combined with functional assays for precise antigen identification, are necessary to fully elucidate the specific roles of the IgG-targeted microbiota in the pathogenesis of UC.

In conclusion, the application of the IPS, which statistically integrates the sample-specific IgG-binding rate to mitigate background abundance bias, enabled the identification of IgG-targeted bacterial populations that could not be characterized based on relative abundance alone. Our in vitro findings suggest that the capacity of systemic IgG to target the gut microbiota is not directed uniformly but is selectively targeted toward specific bacterial taxa depending on the patient’s immunological status. In particular, oral-associated bacteria and mucin-degrading taxa were associated with disease activity and systemic inflammation, identifying them as major targets associated with the aberrant host humoral immune response in this cohort. These findings provide further insight into host–microbiota interactions in UC and offer a novel analytical framework for evaluating candidate immune-targeted bacterial populations associated with disease activity.

## 4. Materials and Methods

### 4.1. Patients

Fecal and serum samples used in this study were collected from 37 patients with UC and 4 healthy participants. Study participants were recruited, and sample collection was conducted between December 2012 and May 2013. Following collection, all serum and fecal samples were immediately processed and stored at −80 °C until the in vitro IgG-binding assays and microbiome analyses were performed for the present study. The baseline characteristics of the study participants are summarized in Table 1. Detailed clinical metadata for the study cohort, including disease duration, disease extent, and medications, are provided in Table S1. The pharmacological treatments received by the UC patients in this cohort were limited to 5-aminosalicylic acid and probiotics; no patients were receiving corticosteroids, immunomodulators, biologics, or antibiotics at the time of sampling.

### 4.2. Flow Cytometric Analysis of IgG-Bound Bacteria

Fecal samples (0.5 g) were suspended in phosphate-buffered saline (PBS, pH 7.4). After allowing large debris to settle, the supernatant was filtered through a 100-μm cell strainer. The filtrate was diluted with PBS to a final fecal concentration of 2 mg/mL, and the bacterial fraction was harvested by centrifugation at 6000× *g* for 5 min at 4 °C. The resulting pellet was resuspended in 4% paraformaldehyde (PFA) in PBS and stored at 4 °C for fixation until subsequent analysis. The fixed fecal bacterial fraction (equivalent to 20 mg of feces) was washed with PBS, resuspended and thoroughly blocked in 1% bovine serum albumin (BSA)/PBS to prevent non-specific binding, and incubated with autologous patient serum (diluted 1:1000) for 1 h at room temperature. After washing with PBS, an APC-conjugated anti-human IgG (Fc-specific) antibody was added, and the mixture was incubated for 30 min at room temperature. Following another PBS wash, propidium iodide (PI) was added, and the samples were stained on ice for 15 min. All incubations subsequent to the serum reaction were performed in the dark. The prepared samples were analyzed using a flow cytometer (Guava easyCyte 6-2L, MilliporeSigma, Burlington, MA, USA). As shown in Figure S1, the gating strategy involved the initial exclusion of large, complex debris based on forward scatter (FSC) and side scatter (SSC) characteristics, using an *Escherichia coli* culture as a reference. Subsequently, 20,000 PI-positive events were acquired. Because the 4% PFA fixation permeabilizes bacterial cell membranes, PI effectively intercalates into the nucleic acids of all bacterial cells, serving as a reliable total bacterial marker. The threshold for PI positivity was rigorously established using an unstained control. Within this PI-positive gate, events positive for APC were quantified. To define the threshold for true APC positivity and account for non-specific binding of the secondary antibody, a negative control omitting the autologous patient serum (secondary antibody-only control) was processed in parallel for each sample. The percentage of APC-positive events detected in this secondary antibody-only control was subtracted from the corresponding patient sample data to calculate the specific IgG-bound bacterial fraction. Flow cytometric measurements were performed with technical replicates (*n* = 2 per sample).

### 4.3. Purification of Autologous Serum IgG

Frozen serum samples from UC patients were thawed, heat-inactivated at 57 °C for 30 min, and incubated overnight at 4 °C. Following centrifugation at 10,000× *g* for 5 min at 4 °C, 200 μL of the serum fraction was collected for IgG purification. IgG was purified using Micro Bio-Spin Chromatography Columns (Bio-Rad Laboratories, Hercules, CA, USA) packed with Protein G Sepharose 4 Fast Flow (GE Healthcare, Chicago, IL, USA). The serum was loaded onto the column pre-equilibrated with PBS, incubated for 5 min at room temperature, and then washed with PBS. Bound IgG was eluted using 0.1 M Glycine-HCl (pH 2.7) and immediately neutralized with 1 M Tris-HCl (pH 9.5). The eluate was concentrated and buffer-exchanged into PBS via centrifugal ultrafiltration using a Microcon YM-30 (MilliporeSigma, Burlington, MA, USA). The recovered purified IgG solution was centrifuged at 20,000× *g* for 5 min to remove aggregates. The protein concentration of the resulting supernatant was determined using the BCA assay, and the solution was adjusted to a final concentration of 5 mg/mL with PBS prior to subsequent analyses.

### 4.4. Reaction and Magnetic Recovery of IgG-Bound Fecal Bacteria

Fecal samples (0.2 g) were suspended in PBS, and large debris was removed by two repeated low-speed centrifugations (200× *g*, 4 °C, 5 min). The resulting supernatant was centrifuged at 10,000× *g* for 5 min at 4 °C, and the harvested bacterial fraction was washed three times with PBS. This bacterial fraction was resuspended in PBS to a final fecal concentration of 0.1 g/mL. Purified autologous serum IgG (100 μg) was added to the bacterial suspension, and the mixture was gently incubated with end-over-end rotation for 30 min at 4 °C. After the reaction, bacteria were recovered by centrifugation at 10,000× *g* for 5 min at 4 °C and washed a total of four times with PBS. Subsequently, 10 μL of Protein G Mag Sepharose Xtra (GE Healthcare, Chicago, IL, USA) was added to the resulting pellet, and the mixture was incubated with rotation for 30 min at 4 °C. Finally, the supernatant was removed using a magnetic stand, and the beads were magnetically washed six times using 1 mL of PBS per wash to isolate and recover the IgG-bound bacterial fraction. DNA extraction from the recovered beads (IgG-bound bacterial fraction) was performed using the PowerViral Environmental RNA/DNA Isolation Kit (QIAGEN, Hilden, Germany) according to the manufacturer’s protocol to purify DNA for 16S rRNA gene analysis. The concentration of the extracted DNA was quantified using the Qubit dsDNA BR Assay Kit (Thermo Fisher Scientific, Waltham, MA, USA). In this study, the IgG-bound fraction and the unfractionated whole feces were subjected to 16S rRNA gene sequencing. The IgG-negative (unbound) fraction was not sequenced. The analytical framework for evaluating IgG targeting utilized in this study is based on a posterior probability approach modeled on the method by Jackson et al., which mathematically negates the necessity of sequencing the antibody-unbound fraction [33].

### 4.5. 16S rRNA Gene Analysis

Sequencing libraries were prepared by amplifying the V3-V4 hypervariable region of the 16S rRNA gene using the 341F and 805R primer set with Illumina MiSeq adaptor sequences, as previously reported [37]. Libraries were sequenced on the Illumina MiSeq platform using 300-bp paired-end V3 chemistry (Illumina, San Diego, CA, USA) with the addition of 5% PhiX. Sequence denoising and the generation of amplicon sequence variants (ASVs) were performed using the DADA2 plugin within QIIME2. Taxonomic assignment of the resulting ASVs was conducted using a pre-trained Naive Bayes classifier based on the SILVA database (release 138).

### 4.6. Calculation of the IgG+ Probability Score (IPS)

To evaluate the extent of IgG targeting for each bacterial taxon while accounting for its baseline relative abundance, we calculated the IgG+ Probability Score (IPS) using an adjusted formulation based on the approach described by Jackson et al. [33]. Simple ratio-based indices are susceptible to compositional bias, in that low-abundance taxa in the input feces cause mathematical inflation. To mitigate this, for each taxon (ASV), the adjusted IgG+ abundance (*A*_adj_) was calculated by multiplying its relative abundance in the IgG+ fraction by the sample-specific overall IgG binding rate derived from flow cytometry. The denominator (*D*) was defined as the maximum of its relative abundance in the unfractionated whole feces and *A*_adj_. This upper-limit clipping anchors the maximum possible score to the overall IgG binding rate of the sample and prevents inflation driven by rare taxa. A pseudocount of 10^−6^ was added to both the numerator and the denominator to avoid division by zero. The IPS was calculated as follows:

$$A_{{IgG}^{+}}=Abundance\left({IgG}^{+}\right),A_{Feces}=Abundance\left(Feces\right),R_{IgG}=IgG\;binding\;rate$$

$$A_{adj}=A_{{IgG}^{+}}\;\times \;\frac{R_{IgG}}{100}$$

$$D=\max\left(A_{Feces},A_{adj}\right)$$

$$IPS=\frac{A_{adj}+10^{-6}}{D+10^{-6}}$$

where Abundance(IgG+) and Abundance(Feces) represent the relative abundance (%) of a specific taxon in the IgG+ fraction and the whole feces fraction, respectively.

To determine whether the IPS was unduly influenced by the sample-specific overall IgG binding rate, we performed a sensitivity analysis using an alternative metric, the IgG Coating Index (ICI), which does not incorporate the overall IgG binding rate. The ICI was calculated for each taxon as the simple ratio of its relative abundance in the IgG+ fraction to that in the corresponding unfractionated whole feces, with a pseudocount of 10^−6^ added to both terms:

$$ICI=\left(A_{{IgG}^{+}}+10^{-6}\right)/\left(A_{Feces}+10^{-6}\right)$$

The taxonomic associations derived from the ICI were compared with those derived from the IPS (Figure S3).

### 4.7. Predictive Functional Profiling

To predict the functional potential of the gut microbiota, we utilized the PICRUSt2 (Phylogenetic Investigation of Communities by Reconstruction of Unobserved States 2) plugin implemented within the QIIME2 environment. The analysis pipeline was executed using the representative sequences and feature tables derived from the 16S rRNA amplicon sequencing data as input, generating predicted abundance profiles of MetaCyc metabolic pathways for each sample. The resulting profile data were subjected to downstream analyses (calculation of functional IPS and statistical comparisons) in the R environment.

### 4.8. Statistical Analysis

Statistical analyses were performed in the R software environment (v.4.4.2). Microbiome data processing and diversity calculations were conducted using the phyloseq (v.1.50.0) and MicrobiotaProcess (v.1.18.0) packages. The Wilcoxon test was used for intergroup comparisons of α-diversity. β-diversity (Bray–Curtis dissimilarity) between samples was calculated based on the relative abundance of observed ASVs using the vegan package (v.2.7-2) and visualized via principal coordinate analysis (PCoA). Differences in community structure were statistically evaluated by permutational multivariate analysis of variance (PERMANOVA, 9999 permutations) using the adonis function within the same package. The Wilcoxon signed-rank test was used for paired comparisons between the whole feces fraction and the autologous serum IgG-bound (IgG+) fraction, whereas the Wilcoxon rank-sum test (Mann–Whitney U test) was applied for comparisons between two independent groups (e.g., active phase vs. remission).

The IPS, a quantitative metric of IgG targeting, was calculated for each taxon. A functional IPS was similarly calculated for the metabolic pathway profiles predicted by PICRUSt2, and specific enrichment was evaluated by log2 fold change between groups and the Wilcoxon rank-sum test. Continuous correlations between clinical parameters (e.g., CAI, CRP, IgE) and the IgG binding rate (Figure 1) or IPS values (Figure 6) were evaluated using Spearman’s rank correlation analysis and visualized with network plots (corrr package, v.0.4.5) and heatmaps (pheatmap package, v.1.0.13). Independent associations between clinical metadata and bacterial taxa were analyzed using the MaAsLin2 package (v.1.20.0). Multivariate models were constructed incorporating potential confounding factors, such as age, sex, and disease activity, as covariates. The generated regression coefficients were used to evaluate the direction and effect size of the associations. A machine learning algorithm using the randomForest package (v.4.7-1.2) was applied to construct exploratory classification models for clinical phenotypes (disease activity, CAI, and CRP) based on the IPS profiles. Variable importance was evaluated based on the Mean Decrease in Gini impurity. To construct exploratory candidate taxon panels, we initially selected the top 15 taxa (Target Panel) from the unbiased all-taxa model. Subsequently, we performed a feature selection optimization by iteratively calculating the area under the curve (AUC) across varying numbers of top-ranked taxa (from 1 to 15) to identify the smallest “Mini Panel” that achieved plateau performance with the minimum number of predictive features. The exploratory discriminatory capacity of these models was evaluated by receiver operating characteristic (ROC) analysis using the pROC package (v.1.19.0.1), and the AUC and 95% confidence intervals based on the bootstrap method were calculated. Specifically, the Random Forest models were built using 500 trees (ntree = 500), with the number of variables sampled at each split (mtry) set to the default square root of the total number of features. To ensure reproducibility, a random seed was set (set.seed(123)). No explicit class balancing procedures were applied. Importantly, because feature selection and model evaluation were performed on the full dataset without nested cross-validation, the resulting AUCs represent exploratory discriminatory capacity within this specific cohort and should not be interpreted as validated diagnostic performance.

For analyses involving multiple comparisons, such as those performed with MaAsLin2, *p*-values were adjusted to the false discovery rate (FDR) using the Benjamini–Hochberg method to control for false positives. Across all statistical tests, a nominal two-sided *p*-value or an FDR-adjusted *p*-value (*q*-value) of <0.05 was considered statistically significant, as specified in the respective analyses.

## Figures and Tables

**Table 1:** Clinical characteristics of the UC patients.

Category	Disease Activity			Disease Extension			
	Active Period	Remission Period	*p*-Value ^3^	Pancolitis	Ulcerative Proctitis	Left-Sided Colitis	*p*-Value ^3^
No. of subjects	24	13	-	18	9	10	-
Age ^1^	41.5 (26–73)	47 (24–75)	0.239	40 (27–73)	47 (32–65)	38 (24–75)	0.477
Gender (Male: Female)	11:13	6:7	1.00 *	10:8	3:6	4:6	0.578 *
CAI score ^1^	3 (1–15)	0 (0–1)	0.002	1 (0–6)	0 (0–15)	4 (0–8)	0.139
IgG binding rate ^2^	21.1 (0.4–89.7)	10.7 (1.3–26)	0.272	17.6 (0.4–65.3)	9.48 (1.3–18.4)	24.1 (1.8–89.7)	0.461
CRP ^2^	0.203 (0.01–1.1)	0.153 (0.01–1.7)	0.875	0.184 (0.01–1.7)	0.137 (0.01–1.1)	0.232 (0.01–1)	0.333
WBC ^2^	5742 (2400–9400)	4874 (3400–7100)	0.152	5448 (2400–9400)	5370 (3900–7100)	5477 (3900–8300)	0.805
RBC ^2^	441 (352–531)	443 (367–539)	0.899	438 (352–531)	439 (389–479)	449 (371–539)	0.746
PLT ^2^	29.0 (14.6–56)	21.9 (18.1–28.9)	0.003	26.5 (14.6–56)	24.6 (21–33.1)	28.3 (18.1–53.7)	0.966
Hb ^2^	13.0 (9.3–16.1)	14.0 (11.4–17.7)	0.232	13.4 (10.4–17.7)	13.7 (12.1–15.5)	12.9 (9.3–16.9)	0.627
Ht ^2^	39.7 (31.3–50.5)	41.9 (34.4–50.2)	0.364	40.5 (31.3–50.5)	41.4 (37.2–47.8)	39.7 (31.7–50.2)	0.665
ALB ^2^	4.30 (3.7–4.8)	4.49 (4.1–5)	0.116	4.37 (4–4.8)	4.42 (3.9–4.8)	4.31 (3.7–5)	0.623
TCho ^2^	207 (158–239)	200 (148–248)	0.464	207 (158–245)	192 (148–220)	211 (164–248)	0.149
Tbil ^2^	0.571 (0.3–0.9)	0.815 (0.4–1.8)	0.090	0.644 (0.4–1.6)	0.722 (0.4–1.8)	0.620 (0.3–1.1)	0.895
AST ^2^	21.5 (12–42)	20.8 (12–30)	0.975	22.9 (12–42)	18.9 (14–30)	20.3 (14–30)	0.235
ALT ^2^	20.6 (7–74)	17.1 (9–38)	0.975	23.7 (7–74)	14.0 (9–22)	16.5 (7–38)	0.412
ALP ^2^	209 (104–289)	200 (97–306)	0.484	198 (97–289)	200 (104–306)	226 (178–266)	0.154
gGTP ^2^	23.6 (9–104)	23.0 (11–58)	0.599	28.9 (10–104)	15.4 (11–22)	20.5 (9–58)	0.236
LDH ^2^	183 (123–275)	178 (103–281)	0.417	175 (103–225)	195 (130–281)	182 (136–275)	0.793
AMY ^2^	84.3 (53–212)	74.9 (41–106)	0.599	82.6 (41–212)	79.0 (56–106)	79.9 (53–126)	0.903
IgA ^2^	246 (132–373)	261 (166–457)	0.645	273 (139–457)	226 (166–289)	234 (132–373)	0.179
IgG ^2^	1471 (1074–2093)	1289 (973–1791)	0.083	1481 (1086–2093)	1241 (973–1648)	1421 (976–2064)	0.156
IgE ^2^	294 (4–1588)	148 (15–966)	0.227	313 (4–1297)	276 (15–1588)	86.9 (14–450)	0.126
IgM ^2^	112 (44–240)	107 (48–190)	0.911	101 (44–237)	100 (48–190)	137 (62–240)	0.176

^1^ Data are expressed as median (range). ^2^ Data are expressed as mean (range). ^3^ Statistical significance between two groups (Disease activity) was evaluated using the Wilcoxon rank-sum test (Mann–Whitney U test). Differences among three groups (Disease extension) were analyzed using the Kruskal–Wallis test. A *p*-value < 0.05 was considered statistically significant. * For categorical data (Gender), Fisher’s exact test was performed. Abbreviations: CAI, Clinical Activity Index; CRP, C-reactive protein; WBC, White blood cell; RBC, Red blood cell; PLT, Platelets; Hb, Hemoglobin; Ht, Hematocrit; ALB, Albumin; Tcho, Total cholesterol; Tbil, Total bilirubin; AST, Aspartate aminotransferase; ALT, Alanine aminotransferase; ALP, Alkaline phosphatase; γGTP, γ-glutamyl transpeptidase; LDH, Lactate dehydrogenase; AMY, Amylase.

## Data Availability

The 16S rRNA gene sequencing read data have been deposited into DDBJ (Accession number: DRR1082041-DRR1082122). Data used to generate the figures and tables can be provided upon reasonable request to the corresponding author.

## Supplementary Materials

The following supporting information can be downloaded at https://www.mdpi.com/article/10.3390/ijms27209081/s1.

## Abbreviations

The following abbreviations are used in this manuscript:

ALB	Albumin
ALP	Alkaline phosphatase
ALT	Alanine aminotransferase
AMY	Amylase
APC	Allophycocyanin
AST	Aspartate aminotransferase
ASV	Amplicon sequence variant
AUC	Area under the curve
CAI	Clinical Activity Index
CRP	C-reactive protein
FDR	False discovery rate
FSC	Forward scatter
γGTP	γ-glutamyl transpeptidase
Hb	Hemoglobin
HP	Healthy participant
Ht	Hematocrit
IBD	Inflammatory bowel disease
ICI	IgG Coating Index
IgA	Immunoglobulin A
IgE	Immunoglobulin E
IgG	Immunoglobulin G
IgM	Immunoglobulin M
IPS	IgG+ Probability Score
LDH	Lactate dehydrogenase
MaAsLin2	Microbiome Multivariable Association with Linear Models
PCoA	Principal coordinate analysis
PERMANOVA	Permutational multivariate analysis of variance
PFA	Paraformaldehyde
PI	Propidium iodide
PICRUSt2	Phylogenetic Investigation of Communities by Reconstruction of Unobserved States 2
PLT	Platelets
RBC	Red blood cell
ROC	Receiver operating characteristic
SSC	Side scatter
Tbil	Total bilirubin
Tcho	Total cholesterol
UC	Ulcerative colitis
WBC	White blood cell
