# Supplementary material for: Profiling Systemic IgG-Targeted Gut Microbiota Reveals Disease Activity-Associated Bacterial Signatures in Ulcerative Colitis
Source: Int J Mol Sci. 2026 Oct 13;27(20):9081. doi: 10.3390/ijms27209081 (PMC13705514; doi:10.3390/ijms27209081)
Supplement: Supplementary file 1 [file ijms-27-09081-s001.zip › Suppl. Figures S1-S4.pdf]

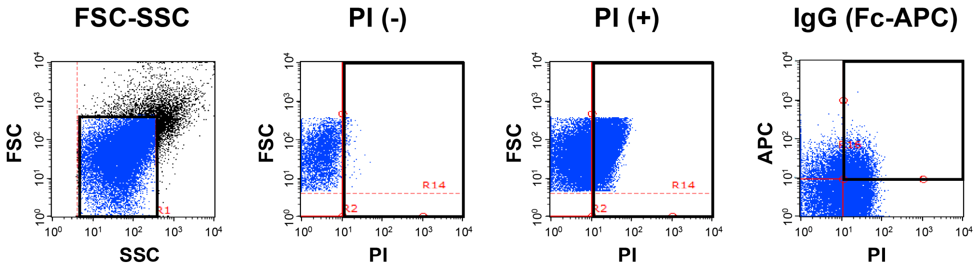

Figure S1. Flow cytometric gating strategy for the identification of IgG-bound fecal bacteria. Representative flow cytometry plots illustrating the sequential gating strategy. **(First panel, FSC-SSC)**: Large, complex debris was excluded based on forward scatter (FSC) and side scatter (SSC) characteristics, using an *Escherichia coli* culture as a reference, to gate the putative bacterial population (R1). **(Second panel, PI (-))**: An unstained control was utilized to accurately establish the threshold for propidium iodide (PI) fluorescence (R14 gate). **(Third panel, PI (+))**: The sample stained with PI. Because 4% PFA fixation permeabilizes the bacterial cell membranes, PI stains the nucleic acids of all bacterial cells. The PI-positive population (R14) was defined as the total bacterial fraction. **(Fourth panel, IgG (Fc-APC))**: Within the PI-positive total bacterial gate, the subpopulation positively stained with the APC-conjugated anti-human IgG antibody was quantified as IgG-bound bacteria (upper-right quadrant). A secondary antibody-only control (omitting patient serum) was processed in parallel to establish the APC positivity threshold and subtract non-specific background fluorescence.

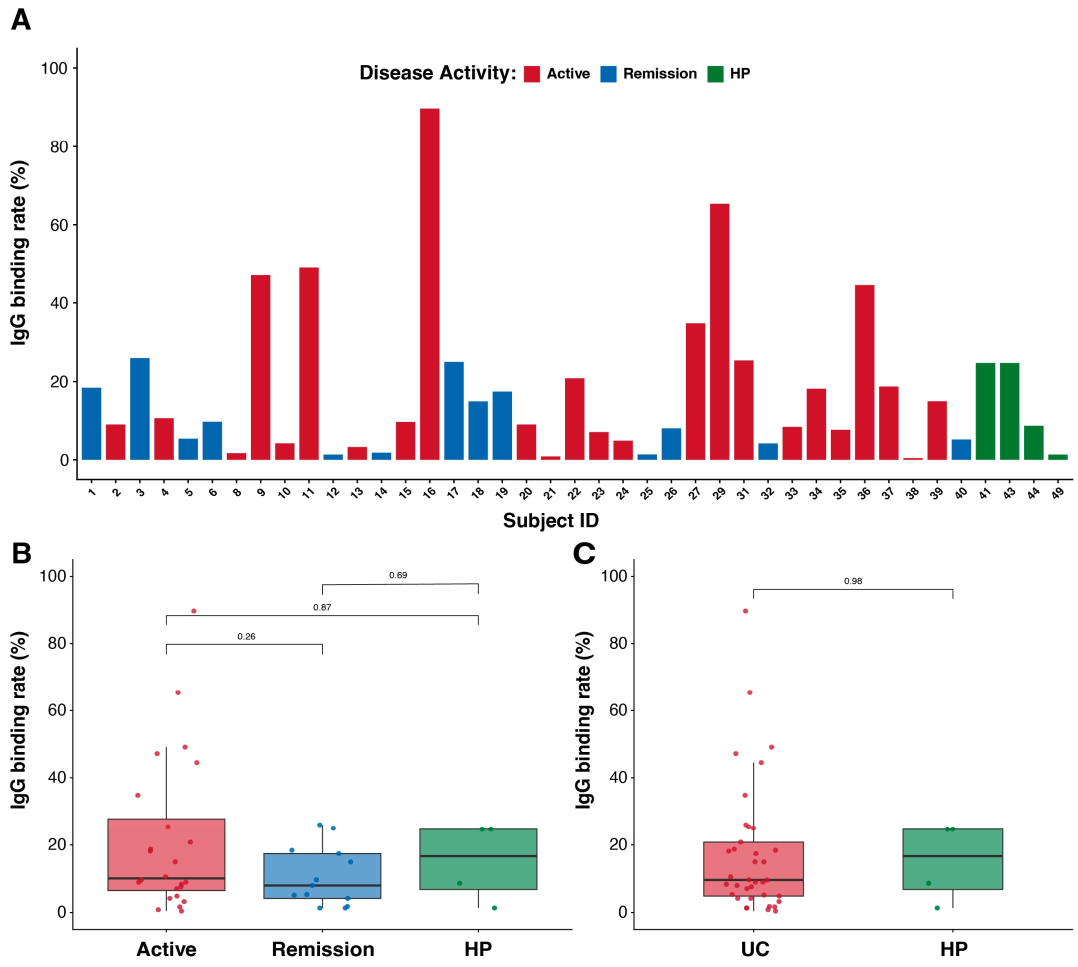

Figure S2. Evaluation of autologous serum IgG binding rates against the gut microbiota in the ulcerative colitis cohort.

(**A**) Distribution of the IgG binding rate (%) against gut bacteria across all samples used in this study. Fecal samples fixed with 4% PFA were reacted with autologous patient sera *in vitro*, followed by staining with an APC-conjugated anti-human IgG Fc antibody and the nucleic acid stain propidium iodide (PI). Among the events within the FSC/SSC gate, the fraction exhibiting both PI and APC positivity was quantified as the percentage of IgG-bound gut bacteria.

(**B**) Comparison of the IgG binding rate (%) among three groups: healthy participants (HP, $n = 4$), UC patients in remission (Remission, $n = 13$), and UC patients in the active phase (Active, $n = 24$). Data are presented as box plots (indicating the median and interquartile range), overlaid with dots representing individual measurements. Statistical significance was evaluated using the Kruskal-Wallis test for an overall comparison across the three groups, followed by pairwise Wilcoxon rank-sum tests for *post hoc* comparisons.

(**C**) Comparison of the IgG binding rate (%) between the healthy participant group (HP, n = 4) and the total UC patient cohort (UC, n = 37). Statistical significance was evaluated using the Wilcoxon rank-sum test (Mann-Whitney U test).

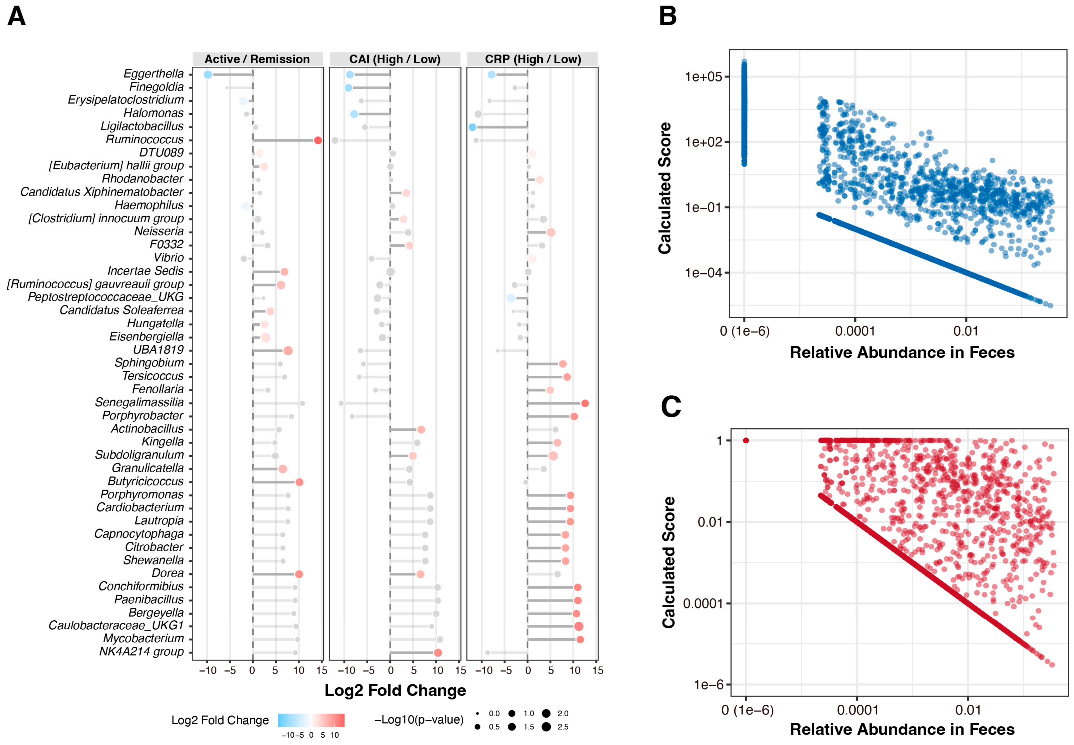

Figure S3. Sensitivity analysis comparing the IgG+ Probability Score (IPS) with a simple ratio-based normalization metric. (A) Variations in the IgG Coating Index (ICI) of gut bacterial taxa based on clinical parameters. Log2 fold changes of the ICI for each bacterial taxon between the two groups in the UC cohort are shown. The ICI was calculated as a simple ratio of relative abundance in the IgG-bound (IgG+) fraction to that in the whole feces fraction, without incorporating the overall IgG-binding rate. A positive log2 fold change (right side) indicates a higher ICI in the corresponding exacerbation or high-value group, whereas a negative value (left side) indicates a higher ICI in the control (remission or low-value) group. The criteria used for the dichotomous stratification are: disease activity (active vs. remission), Clinical Activity Index (CAI; high > 3 vs. low ≤ 3), and C-reactive protein (CRP; high > 0.3 mg/dL vs. low ≤ 0.3 mg/dL). Statistical significance between groups was evaluated using the Wilcoxon rank-sum test, with the size of each dot reflecting the level of significance (-log10 nominal *p*-value). Taxa exhibiting statistically significant variations (nominal $p < 0.05$ and |log2 fold change| ≥ 1) are highlighted in colors corresponding to their direction of change.

(B) Scatter plot of the simple ratio-based index (ICI). The y-axis represents the calculated score, defined as the relative abundance in the IgG+ fraction divided by the relative abundance in unfractionated feces, plotted against the relative abundance in unfractionated feces on the x-

axis. When the relative abundance in feces is low (near the detection limit of 1e-6), technical noise in the IgG+ fraction causes mathematical inflation, generating outliers (>10^2 to 10^5).

(C) Scatter plot of the IgG+ Probability Score (IPS). The y-axis represents the calculated IPS, plotted against the relative abundance in unfractionated feces on the x-axis. The IPS incorporates an upper-limit clipping step that uses the maximum value between the unfractionated feces abundance and the adjusted IgG abundance in the denominator. This step anchors the maximum possible score to the overall IgG binding rate of the sample, preventing the low-abundance inflation observed in Panel B and stabilizing the values for compositional data.

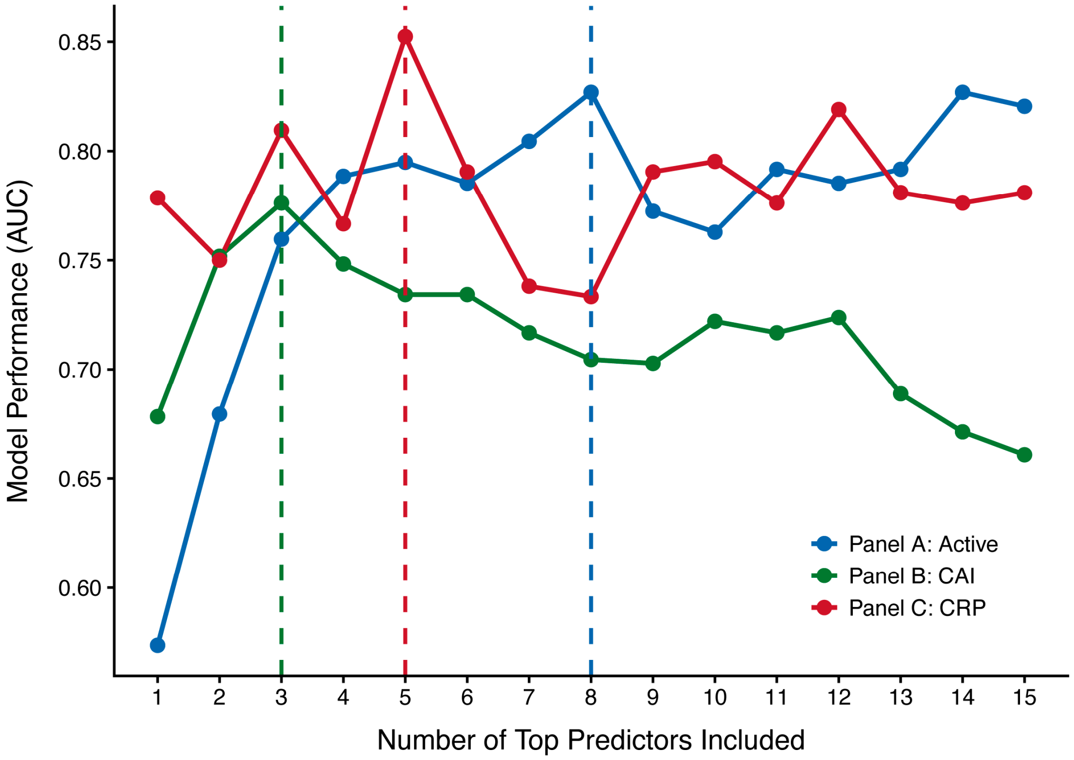

Figure S4. Determination of the optimal number of predictor variables for the Random Forest predictive models.

Feature selection curves evaluating the relationship between the number of predictors incorporated into the models and the resulting predictive accuracy (AUC), utilizing the key bacterial taxa extracted from the Target IPS Panel (**Fig. 8**). The x-axis represents the number of taxa sequentially added to the model in descending order of variable importance (Mean Decrease Gini), while the y-axis denotes the area under the receiver operating characteristic curve (AUC) of the constructed models. The blue (A), green (B), and red (C) lines illustrate the trajectories of predictive accuracy for models targeting disease exacerbation (Active), clinical disease activity (CAI), and systemic inflammation (CRP), respectively. The colored vertical dashed lines indicate the optimal number of variables at which the AUC is maximized for each prediction target. The top 8 predictors for disease exacerbation prediction, the top 3 for CAI prediction, and the top 5 for CRP prediction were extracted and adopted as the final optimized predictive models (Top Mini Panel).
